# Correction: Scalable methods for analyzing and visualizing phylogenetic placement of metagenomic samples

**DOI:** 10.1371/journal.pone.0219925

**Published:** 2019-07-11

**Authors:** The PLOS ONE Staff

## Notice of Republication

This article was republished on July 2, 2019 to correct for missing units of measurement that were incorrectly removed during the typesetting process. The publisher apologizes for these errors. Please download this article again to view the correct version. The originally published, uncorrected article and the republished, corrected article are provided here for reference.

## Supporting information

S1 File Originally published, uncorrected article. (PDF)

S2 File Republished, corrected article. (PDF)
