# Supplementary material for: Scalable methods for analyzing and visualizing phylogenetic placement of metagenomic samples
Source: PLoS One. 2019 May 28;14(5):e0217050. doi: 10.1371/journal.pone.0217050 (PMC6538146; doi:10.1371/journal.pone.0217050)
Supplement: S1 Text — (PDF) [file pone.0217050.s001.pdf]

# S1 Text: Empirical datasets

## Scalable methods for analyzing and visualizing phylogenetic placement of metagenomic samples

Lucas Czech and Alexandros Stamatakis

The analyses and figures presented in the manuscript were conducted on distinct reference alignments and trees, suited for each dataset. Firstly, for the Bacterial Vaginosis (BV) dataset, we used the set of reference sequences from the original study [1], and re-inferred a tree on them. Secondly, for the Tara Oceans (TO) and Human Microbiome Project (HMP) datasets, we used our Phylogenetic Automatic (Reference) Tree (PhAT) method [2] to construct sets of suitable reference sequences from the Silva database [3, 4]. We used the 90% threshold consensus sequences; see [2] for details.

For all analyses, we used the following software setup: Unconstrained maximum likelihood trees were inferred using RAxML v8.2.8 [5]. For aligning reads against reference alignments and reference trees, we used a custom MPI wrapper for PaPaRa 2.0 [6, 7], which is available at [8]. We then applied the `chunkify` procedure as explained in [2] to split the sequences into chunks of unique sequences prior to conducting the phylogenetic placement, in order to minimize processing time. Phylogenetic placement was conducted using EPA-ng [9, 10], which is a faster and more scalable phylogenetic placement implementation than RAxML-EPA [11] and pplacer [12]. Lastly, given the per-chunk placement files produced by EPA-ng, we executed the `unchunkify` procedure of [2] to obtain per-sample placement files. These subsequently served as the input data for the methods presented here.

We made the scripts, data and other tools used for the tests and figures presented here available at http://github.com/lczech/placement-methods-paper. See there for further details.

## 1 Bacterial Vaginosis

We used the Bacterial Vaginosis dataset [1] in order to compare our novel methods to existing ones such as Edge PCA and Squash Clustering [13, 14]. The dataset contains metabarcoding sequences of the vaginal microbiome of 220 women, and was kindly provided by Sujatha Srinivasan. This small dataset with a total of 426 612 query sequences, thereof 15 060 unique, was already analyzed with phylogenetic placement methods in the original publication [1] and in [13]. We re-inferred the reference tree of the original publication using the original alignment, which contains 797 reference sequences specifically selected to represent the vaginal microbiome. As the query sequences were already prepared, no further preprocessing was applied prior to phylogenetic placement. The available per-sample quantitative meta-data for this dataset comprises the Nugent score [15], the value of Amsel's criteria [16], and the vaginal pH value. We used all three meta-data types in our analyses.

For our comparison of Placement-Factorization to the original Phylofactorization [17], we furthermore conducted OTU clustering of the sequences, using two different methods: We used vsearch v2.9.1 [18] as well as swarm v2.2.2 [19, 20] to obtain two sets of OTU clusters. We filtered the OTU table to remove low abundance OTUs, by only keeping those that appear in more than 10% of the samples. In order to assign each OTU to a fitting taxonomic path, we used the assign command of our toll gappa. To this end, we placed the OTUs on the BV reference tree mentioned above, in order to obtain taxonomic assignments for the OTUs that are in line with the taxonomic labels used in our other analyses of the dataset. Each set of OTUs was subsequently aligned with MAFFT v7.310 [21, 22], using the L-INS-i strategy [23]. Finally, we inferred an OTU tree for each set, using the recent RAxML-NG v0.7.0 [24]. These two OTU trees were then used with the meta-data for conducting an analysis with Phylofactor, based on the excellent tutorials at https://github.com/reptalex/phylofactor. The results for the first ten factors for each of these two trees is for example shown in S3 Table of the supplement.

## 2 Tara Oceans

The Tara Oceans (TO) dataset [25, 26, 27] that we used here contains amplicon sequences of protists, and is available at https://www.ebi.ac.uk/ena/data/view/PRJEB6610. At the time of download, there were 370 samples available with a total of 49 023 231 sequences. As the available data are raw `fastq` files, we followed [28] to generate cleaned per-sample `fasta` files. For this, we used our tool PEAR [29] to merge the paired-end reads; CUTADAPT [30] for trimming tags as well as forward and reverse primers; and VSEARCH [18] for filtering erroneous sequences and generating per-sample `fasta` files. We filtered out sequences below 95 bps and above 150 bps, to remove potentially erroneous sequences. No further preprocessing (such as chimera detection) was applied. This resulted in a total of 48 036 019 sequences, thereof 27 697 007 unique. The sequences were then used for phylogenetic placement as explained above. We placed the sequences on the unconstrained *Eukaryota* reference tree obtained via our Phylogenetic Automatic (Reference) Trees (PhAT) method [2], which comprises 2059 taxa, thereof 1795 eukaryotic sequences. The remaining 264 taxa are *Archaea* and *Bacteria*, and were included as a broad outgroup. The TO dataset has a rich variety of per-sample meta-data features; in the context of this paper, we mainly focus on quantitative features such as temperature, salinity, as well as oxygen, nitrate and chlorophyll content of the water. Furthermore, each sample has meta-data features indicating the date, longitude and latitude, depth, etc. of the sampling location. This data might be interesting for further correlation analyses based on geographical information. We did not use them here, as for example longitude and latitude would require a more involved method that also accounts for, e.g., ocean currents. Furthermore, geographical coordinates yield pairwise distances between samples, which are not readily usable with our correlation analysis. Lastly, in order to use features such as the date, that is, in order to analyze samples over time, the same sampling locations would need to be visited at different times during the year, which is not the case for the Tara Oceans expedition.

## 3 Human Microbiome Project

We used the Human Microbiome Project (HMP) dataset [31, 32] for testing the scalability of our methods. In particular, we used the "HM16STR" data of the initial phase "HMP1", which are available from http://www.hmpdacc.org/hmp/. The dataset consists of trimmed 16S rRNA sequences of the `V1V3`, `V3V5`, and `V6V9` regions. The data are further divided into a "by_sample" set and a "healthy" set, which we merged in order to obtain one large dataset, with a total of 9811 samples. We then removed 10 samples that were larger than 70 MB as well as 605 samples that had fewer than 1500 sequences, because we considered them as defective or unreliable outliers. Finally, we also removed 2 samples that did not have a valid body site label assigned to them. This resulted in a set of 9192 samples containing a total of 118 702 967 sequences with an average length of 413 bps. From these samples, sequences with a length of less than 150 bps as well as sequences longer than 540 bps were removed, as we considered them potentially erroneous. No further preprocessing (such as chimera detection) was applied. This resulted in a total of 116 520 289 sequences, of which 63 221 538 were unique. We then used the unconstrained *Bacteria* tree of our Phylogenetic Automatic (Reference) Trees (PhAT) method [2] for phylogenetic placement. The tree comprises 1914 taxa, thereof 1797 bacterial sequences. The remaining 117 taxa are *Archaea* and *Eukaryota*, and were included as a broad outgroup. Each sample is labeled with one of 18 human body site locations where it was sampled. This is the only publicly available meta-data feature.

For our re-analysis of the oral/fecal dataset of the original Phylofactorization [17], we used the test data provided at https://github.com/reptalex/phylofactor. We modified the scripts to produce 10 factors instead of the default of using their stopping criterion, in order to be comparable to our implementation and results. For the comparison with our Placement-Factorization, we selected a suitable oral/fecal subset of the HMP dataset as described in the main text.

## References


[1] Srinivasan S, Hoffman NG, Morgan MT, Matsen FA, Fiedler TL, Hall RW, et al. Bacterial communities in women with bacterial vaginosis: High resolution phylogenetic analyses reveal relationships of microbiota to clinical criteria. PLOS ONE. 2012;7(6):e37818. doi:10.1371/journal.pone.0037818.

[2] Czech L, Barbera P, Stamatakis A. Methods for Automatic Reference Trees and Multilevel Phylogenetic Placement. Bioinformatics. 2018; p. 299792. doi:10.1093/bioinformatics/bty767.

[3] Quast C, Pruesse E, Yilmaz P, Gerken J, Schweer T, Yarza P, et al. The SILVA ribosomal RNA gene database project: improved data processing and web-based tools. Nucleic Acids Research. 2013;41(D1):D590–D596. doi:10.1093/nar/gks1219.

[4] Yilmaz P, Parfrey LW, Yarza P, Gerken J, Pruesse E, Quast C, et al. The SILVA and "All-species Living Tree Project (LTP)" taxonomic frameworks. Nucleic Acids Research. 2014;42(D1):D643–D648. doi:10.1093/nar/gkt1209.

[5] Stamatakis A. RAxML version 8: a tool for phylogenetic analysis and post-analysis of large phylogenies. Bioinformatics. 2014;30(9):1312–1313. doi:10.1093/bioinformatics/btu033.

[6] Berger S, Stamatakis A. Aligning short reads to reference alignments and trees. Bioinformatics. 2011;27(15):2068–2075. doi:10.1093/bioinformatics/btr320.

[7] Berger S, Stamatakis A. PaPaRa 2.0: A Vectorized Algorithm for Probabilistic Phylogeny-Aware Alignment Extension. Heidelberg: Heidelberg Institute for Theoretical Studies; 2012.

[8] Berger S, Czech L. PaPaRa 2.0 with MPI; 2016. Available from: https://github.com/lczech/papara_nt.

[9] Barbera P, Kozlov AM, Czech L, Morel B, Darriba D, Flouri T, et al. EPA-ng: Massively Parallel Evolutionary Placement of Genetic Sequences. Systematic Biology. 2018;doi:10.1093/sysbio/syy054.

[10] Barbera P. EPA-ng – Massively Parallel Phylogenetic Placement of Genetic Sequences; 2017. Online: https://github.com/Pbdas/epa-ng.

[11] Berger S, Krompass D, Stamatakis A. Performance, accuracy, and web server for evolutionary placement of short sequence reads under maximum likelihood. Systematic Biology. 2011;60(3):291–302. doi:10.1093/sysbio/syr010.

[12] Matsen FA, Kodner RB, Armbrust EV. pplacer: linear time maximum-likelihood and Bayesian phylogenetic placement of sequences onto a fixed reference tree. BMC Bioinformatics. 2010;11(1):538. doi:10.1186/1471-2105-11-538.

[13] Matsen FA, Evans SN. Edge principal components and squash clustering: using the special structure of phylogenetic placement data for sample comparison. PLOS ONE. 2011;8(3):1–17. doi:10.1371/journal.pone.0056859.

[14] Evans SN, Matsen FA. The phylogenetic Kantorovich-Rubinstein metric for environmental sequence samples. Journal of the Royal Statistical Society Series B: Statistical Methodology. 2012;74:569–592. doi:10.1111/j.1467-9868.2011.01018.x.

[15] Nugent RP, Krohn MA, Hillier SL. Reliability of diagnosing bacterial vaginosis is improved by a standardized method of gram stain interpretation. Journal of clinical microbiology. 1991;29(2):297–301.

[16] Amsel R, Totten PA, Spiegel CA, Chen KCS, Eschenbach D, Holmes KK. Nonspecific vaginitis: Diagnostic Criteria and Microbial and Epidemiologic Associations. The American Journal of Medicine. 1983;74(1):14–22. doi:10.1016/0002-9343(83)91112-9.

[17] Washburne AD, Silverman JD, Leff JW, Bennett DJ, Darcy JL, Mukherjee S, et al. Phylogenetic factorization of compositional data yields lineage-level associations in microbiome datasets. PeerJ. 2017;5:e2969. doi:10.7717/peerj.2969.

[18] Rognes T, Flouri T, Nichols B, Quince C, Mahé F. VSEARCH: a versatile open source tool for metagenomics. PeerJ. 2016;4:e2584.

[19] Mahé F, Rognes T, Quince C, de Vargas C, Dunthorn M. Swarm: Robust and fast clustering method for amplicon-based studies. PeerJ. 2014;2:1–12. doi:http://dx.doi.org/10.7287/peerj.preprints.386v1.

[20] Mahé F, Rognes T, Quince C, De Vargas C, Dunthorn M. Swarm v2: Highly-scalable and high-resolution amplicon clustering. PeerJ. 2015;.

[21] Katoh K, Misawa K, Kuma K, Miyata T. MAFFT: a novel method for rapid multiple sequence alignment based on fast Fourier transform. Nucleic Acids Research. 2002;30(14):3059–3066. doi:10.1093/nar/gkf436.

[22] Katoh K, Standley DM. MAFFT Multiple Sequence Alignment Software Version 7: Improvements in Performance and Usability. Molecular Biology and Evolution. 2013;30(4):772–780. doi:10.1093/molbev/mst010.

[23] Katoh K, Kuma Ki, Toh H, Miyata T. MAFFT version 5: improvement in accuracy of multiple sequence alignment. Nucleic Acids Research. 2005;33(2):511–518. doi:10.1093/nar/gki198.

[24] Kozlov A, Darriba D, Flouri T, Morel B, Stamatakis A. RAxML-NG: A fast, scalable, and user-friendly tool for maximum likelihood phylogenetic inference. bioRxiv. 2018; p. 447110. doi:10.1101/447110.

[25] Karsenti E, Acinas SG, Bork P, Bowler C, de Vargas C, Raes J, et al. A holistic approach to marine Eco-systems biology. PLoS Biology. 2011;9(10):7–11. doi:10.1371/journal.pbio.1001177.

[26] Sunagawa S, Coelho LP, Chaffron S, Kultima JR, Labadie K, Salazar G, et al. Structure and function of the global ocean microbiome. Science. 2015;348(6237):1–10. doi:10.1126/science.1261359.

[27] Guidi L, Chaffron S, Bittner L, Eveillard D, Larhlimi A, Roux S, et al. Plankton networks driving carbon export in the oligotrophic ocean. Nature. 2016;532(7600):465–470. doi:10.1038/nature16942.

[28] Mahé F. Fred's metabarcoding pipeline; 2016. Available from: https://github.com/frederic-mahe/swarm/wiki/Fred's-metabarcoding-pipeline.

[29] Zhang J, Kobert K, Flouri T, Stamatakis A. PEAR: A fast and accurate Illumina Paired-End reAd mergeR. Bioinformatics. 2014;30(5):614–620. doi:10.1093/bioinformatics/btt593.

[30] Martin M. Cutadapt removes adapter sequences from high-throughput sequencing reads. EMBnetjournal. 2011;17(1):10. doi:10.14806/ej.17.1.200.

[31] Huttenhower C, Gevers D, Knight R, Abubucker S, Badger JH, Chinwalla AT, et al. Structure, function and diversity of the healthy human microbiome. Nature. 2012;486(7402):207–214. doi:10.1038/nature11234.

[32] Methé BA, Nelson KE, Pop M, Creasy HH, Giglio MG, Huttenhower C, et al. A framework for human microbiome research. Nature. 2012;486(7402):215–221. doi:10.1038/nature11209.A.
