# Supplementary material for: Scalable methods for analyzing and visualizing phylogenetic placement of metagenomic samples
Source: PLoS One. 2019 May 28;14(5):e0217050. doi: 10.1371/journal.pone.0217050 (PMC6538146; doi:10.1371/journal.pone.0217050)
Supplement: S2 Text — (PDF) [file pone.0217050.s002.pdf]

S2 Text: Pipeline and Implementation

Scalable methods for analyzing and visualizing phylogenetic placement of metagenomic samples

Lucas Czech and Alexandros Stamatakis

1 Phylogenetic Placement Pipeline

Phylogenetic placement (also called *evolutionary placement*) has been developed for conducting phylogenetic analyses of metagenomic sequence data [1]. It is implemented in tools such as PPLACER [2], RAxML-EPA [3], and EPA-NG [4, 5]. Instead of resolving the phylogeny of a set of metagenomic sequences, phylogenetic placement treats each sequence, called a *query sequence* (QS), separately. It evaluates how these QSs relate to an existing *reference tree* (RT) based on known reference sequences. For each QS, it computes the probabilities of *placing* the sequence on all branches of the RT, thereby classifying them into a phylogenetic context of related sequences, without the need to resolve relationships between the QSs.

In the most common use case, the QSs are reads or amplicons from environmental samples. Most often barcoding regions or marker genes such as 16S or 18S are used, but there also exist studies that use different, or even a set of, marker genes [6]. Furthermore, other types of sequences such as $_{\text{mi}}$tags [7] can be used.

The RT and the reference sequences it represents are typically assembled by the user so that they capture the expected species diversity in the samples. To expedite this process, we recently proposed an automated approach for assembling suitable sets of reference sequences [8]. Distinct samples from one study are typically placed on the same underlying RT in order to facilitate comparisons between the samples.

We here assume to be given a set of suitable reference sequences, their alignment, and an RT inferred from them. In current implementations of phylogenetic placement, the RT has to be strictly bifurcating. Prior to the placement, the QSs need to be aligned against the reference alignment of the RT by programs such as PaPaRa [9, 10] or HMMALIGN [11, 12]. The input to phylogenetic placement are (i) the reference tree (RT), (ii) its underlying alignment, and (iii) the aligned query sequences (QSs). The placement pipeline is shown in Fig S1.

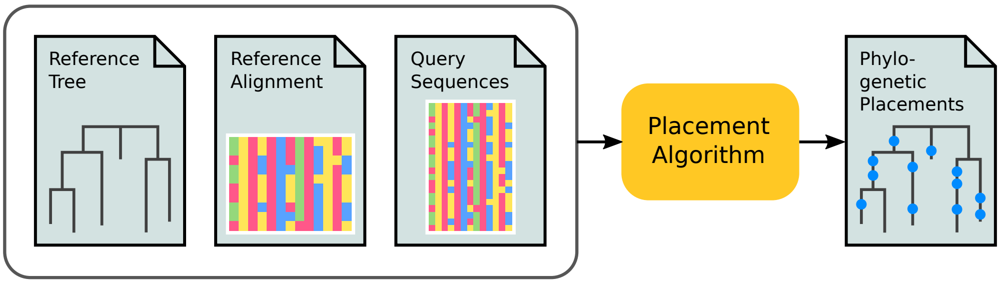

Phylogenetic placement pipeline. The input to phylogenetic placement are three files: the reference tree (RT), the corresponding reference alignment, and the aligned query sequences (QSs). The placement algorithm then computes the probabilities of placing the QSs on the branches of the RT, which are stored in an output file.

The output of phylogenetic placement are the probabilities of placing the QSs on the branches of the RT. The output data is usually stored in so-called `jplace` files [13]. It stores the RT in `Newick` format, including tip names and branch lengths. Its main part is the list of placements for each QS, which store the likelihood weight ratios (LWRs) and placement positions along the branches of the RT.

2 Method Implementation

The methods described in the manuscript are implemented in our tool GAPPA, which is freely available under GPLv3 at http://github.com/lczech/gappa. GAPPA internally uses our C++11 library GENESIS, which offers functionality for working with phylogenies and phylogenetic placement data, and also contains methods to work with taxonomies, sequences and many other data types. GENESIS is also freely available under GPLv3 at http://github.com/lczech/genesis. This software design of using a library (GENESIS) for the core functions, and a separate program (GAPPA) for the user-facing command line interface, has the advantage of enabling experimentation and extension for future research.

GAPPA offers a command line interface for conducting typical tasks when working with phylogenetic placements. The methods that we described here are implemented via the following sub-commands:

- `dispersion`: The command takes a set of jplace files (the samples), and calculates and visualizes the Edge Dispersion per edge of the reference tree.
- `correlation`: The command takes a set of jplace samples, as well as a table containing metadata features for each sample. It then calculates and visualizes the Edge Correlation with the metadata features per edge of the reference tree.
- `phylogenetic-kmeans` and `imbalance-kmeans`: Performs k-means clustering of a set of jplace files according to our methods.
- `placement-factorization`: Performs our adaptation of Phylofactorization [14] to phylogenetic placement data, and outputs all relevant analysis results.
- `squash` and `edgepca`: Reimplementations of the two existing methods [15, 16].

These are the GAPPA commands that are relevant for this paper. The tool also offers additional commands that are useful for phylogenetic placement data, such as visualization or filtering. At the time of writing this manuscript, GAPPA is under active development, with more functions planned in the near future. Furthermore, GAPPA has recently been bundled into a BIOCONDA package, which is available at https://anaconda.org/bioconda/gappa; note that this package is not maintained by ourselves. Lastly, we provide prototype implementations, scripts, data, and other tools used for the tests and figures in this paper at http://github.com/lczech/placement-methods-paper.

References

[1] von Mering C, Hugenholtz P, Raes J, Tringe SG, Doerks T, Jensen LJ, et al. Quantitative Phylogenetic Assessment of Microbial Communities in Diverse Environments. Science. 2007;315(5815):1126–1130. doi:10.1126/science.1133420.

[2] Matsen FA, Kodner RB, Armbrust EV. pplacer: linear time maximum-likelihood and Bayesian phylogenetic placement of sequences onto a fixed reference tree. BMC Bioinformatics. 2010;11(1):538. doi:10.1186/1471-2105-11-538.

[3] Berger S, Krompass D, Stamatakis A. Performance, accuracy, and web server for evolutionary placement of short sequence reads under maximum likelihood. Systematic Biology. 2011;60(3):291–302. doi:10.1093/sysbio/syr010.

[4] Barbera P, Kozlov AM, Czech L, Morel B, Darriba D, Flouri T, et al. EPA-ng: Massively Parallel Evolutionary Placement of Genetic Sequences. Systematic Biology. 2018;doi:10.1093/sysbio/syy054.

[5] Barbera P. EPA-ng – Massively Parallel Phylogenetic Placement of Genetic Sequences; 2017. Online: https://github.com/Pbdas/epa-ng.

[6] Sunagawa S, Mende DR, Zeller G, Izquierdo-Carrasco F, Berger Sa, Kultima JR, et al. Metagenomic species profiling using universal phylogenetic marker genes. Nature Methods. 2013;10(12):1196. doi:10.1038/nmeth.2693.

[7] Logares R, Sunagawa S, Salazar G, Cornejo-Castillo FM, Ferrera I, Sarmento H, et al. Metagenomic 16S rDNA Illumina tags are a powerful alternative to amplicon sequencing to explore diversity and structure of microbial communities. Environmental Microbiology. 2014;16(9):2659–2671. doi:10.1111/1462-2920.12250.

[8] Czech L, Barbera P, Stamatakis A. Methods for Automatic Reference Trees and Multilevel Phylogenetic Placement. Bioinformatics. 2018; p. 299792. doi:10.1093/bioinformatics/bty767.

[9] Berger S, Stamatakis A. Aligning short reads to reference alignments and trees. Bioinformatics. 2011;27(15):2068–2075. doi:10.1093/bioinformatics/btr320.

[10] Berger S, Stamatakis A. PaPaRa 2.0: A Vectorized Algorithm for Probabilistic Phylogeny-Aware Alignment Extension. Heidelberg: Heidelberg Institute for Theoretical Studies; 2012.

[11] Eddy SR. Profile hidden Markov models. Bioinformatics. 1998;14(9):755–763.

[12] Eddy SR. A new generation of homology search tools based on probabilistic inference. In: Genome Informatics. vol. 23. World Scientific; 2009. p. 205–211.

[13] Matsen FA, Hoffman NG, Gallagher A, Stamatakis A. A format for phylogenetic placements. PLoS ONE. 2012;7(2):1–4. doi:10.1371/journal.pone.0031009.

[14] Washburne AD, Silverman JD, Leff JW, Bennett DJ, Darcy JL, Mukherjee S, et al. Phylogenetic factorization of compositional data yields lineage-level associations in microbiome datasets. PeerJ. 2017;5:e2969. doi:10.7717/peerj.2969.

[15] Matsen FA, Evans SN. Edge principal components and squash clustering: using the special structure of phylogenetic placement data for sample comparison. PLOS ONE. 2011;8(3):1–17. doi:10.1371/journal.pone.0056859.

[16] Evans SN, Matsen FA. The phylogenetic Kantorovich-Rubinstein metric for environmental sequence samples. Journal of the Royal Statistical Society Series B: Statistical Methodology. 2012;74:569–592. doi:10.1111/j.1467-9868.2011.01018.x.
