# Supplementary material for: Scalable methods for analyzing and visualizing phylogenetic placement of metagenomic samples
Source: PLoS One. 2019 May 28;14(5):e0217050. doi: 10.1371/journal.pone.0217050 (PMC6538146; doi:10.1371/journal.pone.0217050)
Supplement: S1 Table — The table lists the 19 body site labels used by the Human Microbiome Project (HMP) [16, 17]. We used this dataset to evaluate the applicability of our methods for phylogenetic placement. In order to simplify the visualization in several figures, we summarized some of the labels into eight location regions, as shown in the second column. The last column lists how many samples from each body site were used in our evaluation. (PDF) [file pone.0217050.s003.pdf]

Body Site	Region	Samples
Tongue Dorsum	Mouth (back)	610
Palatine Tonsils	Mouth (back)	599
Throat	Mouth (back)	638
Attached Keratinized Gingiva	Mouth (front)	600
Hard Palate	Mouth (front)	566
Buccal Mucosa	Mouth (front)	597
Saliva	Saliva	529
Supragingival Plaque	Plaque	608
Subgingival Plaque	Plaque	595
Anterior Nares	Airways	541
Left Retroauricular Crease	Skin	596
Right Retroauricular Crease	Skin	604
Left Antecubital Fossa	Skin	290
Right Antecubital Fossa	Skin	328
Stool	Stool	600
Vaginal Introitus	Vagina	292
Mid Vagina	Vagina	298
Posterior Fornix	Vagina	301
Sum		9192
