# Supplementary material for: Scalable methods for analyzing and visualizing phylogenetic placement of metagenomic samples
Source: PLoS One. 2019 May 28;14(5):e0217050. doi: 10.1371/journal.pone.0217050 (PMC6538146; doi:10.1371/journal.pone.0217050)
Supplement: S2 Table — Here we show the effect of per-branch placement binning on the run-time and on the resulting relative error when calculating the pairwise KR distance matrix between samples, by example of the Human Microbiome Project (HMP) [16, 17] dataset. Because of the size of the dataset (9192 samples) and reference tree (1914 taxa), we executed this evaluation in parallel on 16 cores. The first row shows the baseline performance, that is, without binning. When using fewer bins per branch, the run-time decreases, at the cost of slightly increasing the average relative error. Still, even when compressing the placement masses into only one bin per branch (that is, just using per-branch masses), the average relative error of the KR distances is around 1%, which is acceptable for most applications. However, considering that the run-time savings are not substantially better for a low number of bins, we recommend using a relatively large number of bins, e.g., 32 or more. This is because run-times of KR distance calculations also depend on other effects such as the necessary repeated tree traversals. We also conducted these tests on the BV dataset, were the relative error is even smaller. (PDF) [file pone.0217050.s004.pdf]

Bins	Time (h:mm)	Speedup	Relative Δ
-	9:46	1.00	0.000000
256	6:58	1.40	0.000008
128	6:39	1.47	0.000015
64	6:30	1.50	0.000035
32	6:25	1.52	0.000124
16	6:13	1.57	0.000272
8	6:08	1.59	0.000669
4	6:07	1.60	0.002747
2	6:04	1.61	0.004284
1	5:35	1.75	0.011585
