# Supplementary material for: Scalable methods for analyzing and visualizing phylogenetic placement of metagenomic samples
Source: PLoS One. 2019 May 28;14(5):e0217050. doi: 10.1371/journal.pone.0217050 (PMC6538146; doi:10.1371/journal.pone.0217050)
Supplement: S3 Table — We analyzed the BV dataset with the original Phylofactorization, using two different methods for the OTU clustering of the data, namely vsearch [54] and swarm [52, 53]; see S1 Text for details on the preprocessing. Here, we compare our Placement-Factorization of the dataset to these results. As the original implementation does not support taxon weighting, we also do not use it here. The table shows the clades split by the first 10 factors found by each variant. See also S14 Fig for a visualization of the clades found by our adaptation. As shown in S1 Fig and in the original study of the dataset [18], there are multiple different taxa that are associated with BV. That is, there are several clades or branches of our reference tree where the placement mass differs between healthy and sick patients. It is thus expected that a phylo-factorization of these data exhibits some variation in the exact clade found, depending on the preprocessing and exact settings being used. Still, the table shows that—apart from ordering—the factored clades are mostly consistent across variants, and consistent with previous findings. All of the taxa found by the swarm-based Phylofactorization and by our Placement-Factorization, as well as all taxa except some of the Streptococcus found as part of the first factor of the vsearch-based Phylofactorization, were already shown to play important roles for this dataset [18]. The inclusion of Streptococcus in the vsearch variant is due to an inner edge that has a slightly higher value of the objective function than the actually more relevant edges leading to the Lactobacillus clade. We observed a similar behavior of large clades being split with our implementation when using taxon weights, as shown in Fig 10. Lastly, the normalized mutual information [110] between the three variants ranges between 71% and 81%, further showing that they mostly find the same clades. (PDF) [file pone.0217050.s005.pdf]

	Original (vsearch)	Original (swarm)	Placement-Factorization
1	Lactobacillus crispatus, Lactobacillus jensenii, Lactobacillus iners, Lactobacillus coleohominis, Lactobacillus gasseri, Lactobacillus vaginalis, Streptococcus agalactiae, Streptococcus anginosus, Streptococcus gallolyticus, Streptococcus oralis, Aerococcus christensenii	Sneathia sanguinegens, Leptotrichia amnionii	Lactobacillus crispatus, Lactobacillus jensenii, Lactobacillus kalixensis
2	Lactobacillus crispatus	Lactobacillus crispatus	Sneathia sanguinegens, Leptotrichia amnionii
3	Gardnerella vaginalis	Gardnerella vaginalis	Gardnerella vaginalis
4	Leptotrichia amnionii	Atopobium vaginae	Megasphaera
5	Megasphaera	Megasphaera	Lactobacillus crispatus
6	Atopobium vaginae	Eggerthella	Eggerthella
7	Eggerthella	Prevotella bivia, Prevotella amnii	Prevotella timonensis, Prevotella buccalis
8	Sneathia sanguinegens	Prevotella timonensis	Prevotella bivia, Prevotella amnii
9	Prevotella timonensis	BVAB2	Atopobium vaginae
10	Lactobacillus jensenii	Lactobacillus jensenii	Lactobacillus iners
