# Supplementary figures and images for: Scalable methods for analyzing and visualizing phylogenetic placement of metagenomic samples
Source: PLoS One. 2019 May 28;14(5):e0217050. doi: 10.1371/journal.pone.0217050 (PMC6538146; doi:10.1371/journal.pone.0217050)

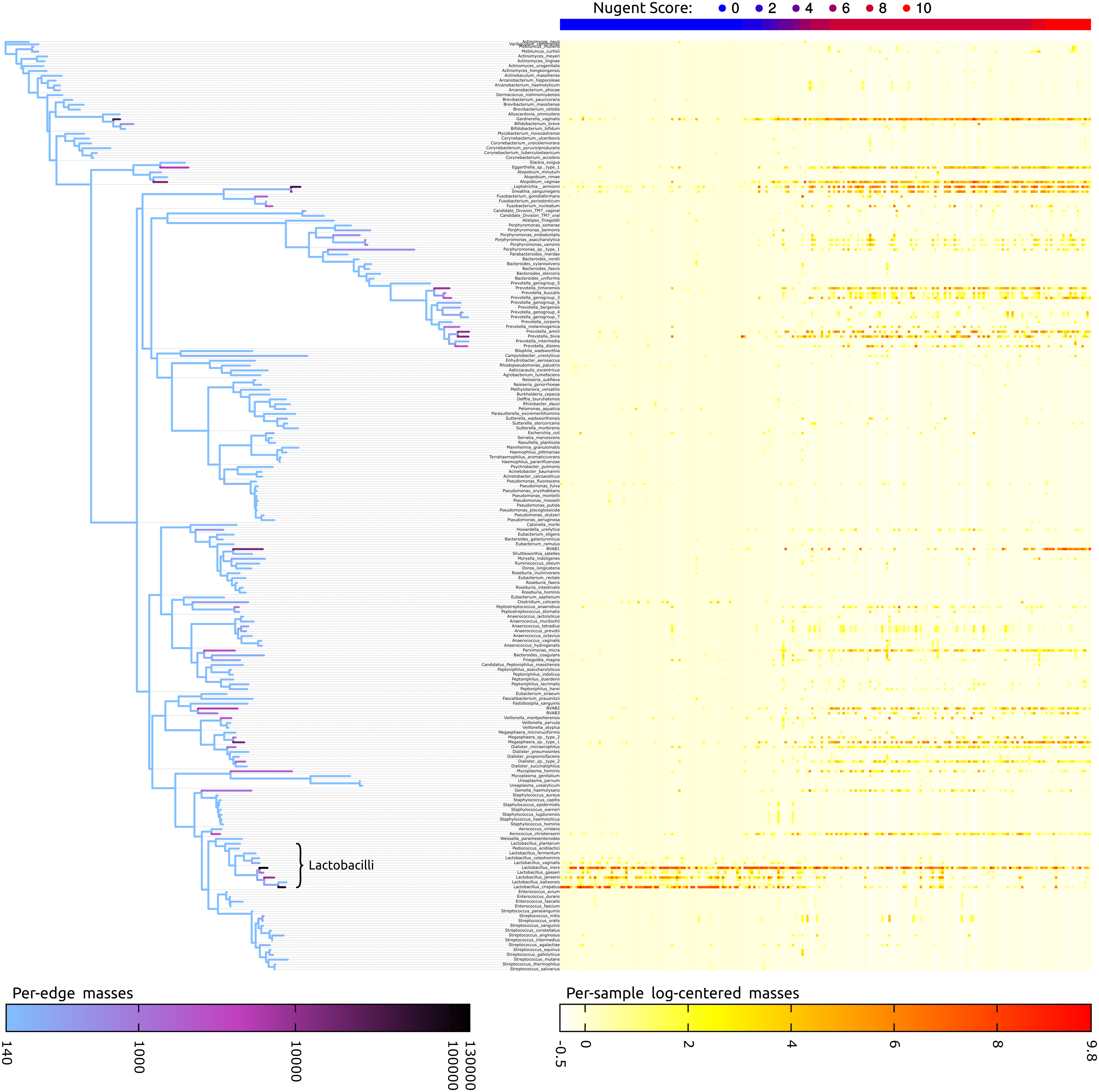

Supplement: S1 Fig — The figure provides an overview of the placement of the BV dataset [18]: The left hand side shows a condensed version of the original reference tree of [18], colored by log-scaled placement mass of all samples accumulated. For clarity and simplicity, in this figure we used a reference tree built from the consensus sequences of each original reference taxon, so that each species is represented by exactly one tip here. The Lactobacillus clade is highlighted in the tree, which is an important clade for this dataset. Note the two particularly dark branches, Lactobacillus iners and Lactobacillus crispatus, which are the major species associated with a healthy vaginal microbiome [18]. The right hand side shows a heat map that further resolves the placement masses per sample: Each row corresponds to a branch of the tree on the left (note that dashed lines also start from inner branches), and each column represents one sample. The values are log-centered in order to be consistent with typical OTU abundance heat map representations, for example as in [31]. The samples/columns are sorted by their Nugent score, from 0 at the left for the healthy patients to 10 at the right for the sick ones. The Nugent score of each sample is also shown at the top as a blue to red bar. Here again the high abundance of Lactobacillus in healthy patients is visible in the lower part of the matrix, while the diseased patients exhibit high placement masses at several other taxa [18]. (TIFF) [file pone.0217050.s006.tiff]

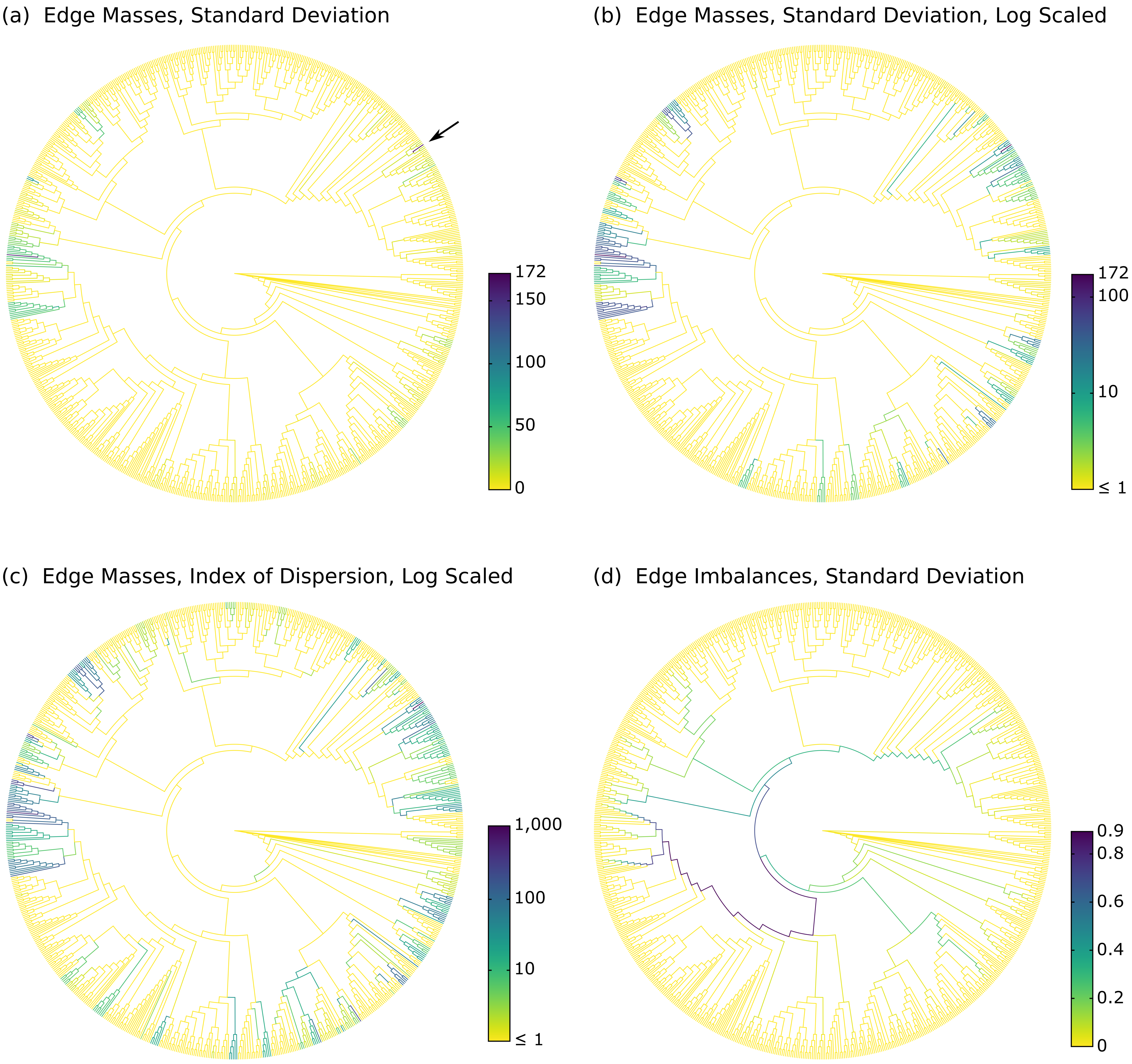

Supplement: S2 Fig — We re-analyzed the BV dataset to show variants of our Edge Dispersion method. All subfigures highlight the same branches and clades as found by other methods such as Edge PCA. The method is useful as a first exploratory tool to detect placement heterogeneity across samples. In contrast to Edge Correlation, it can however not explain the reasons of heterogeneity. Subfigure (a) shows the standard deviation of the absolute edge masses, without any further processing. It is striking that one outlier, marked with an arrow, is dominating, thus hiding the values on less variable edges. This outlier occurs at the species Prevotella bivia in one of the 220 samples, where 2781 out of 2782 sequences in the sample have placement mass on that branch. Upon close examination, this outlier can also be seen in Figure 1D of [18], but is less apparent there. Subfigure (b) is identical to Fig 4(a) of the main text and shows the standard deviation again, but this time using logarithmic scaling, thus revealing more details on the edges with lower placement mass variance. Furthermore, when comparing it to S3(c) Fig, we see that the same clades that exhibit a high correlation or anti-correlation with meta-data there are also highlighted here. Subfigure (c) shows the Index of Dispersion of the edge masses, that is, the variance normalized by the mean. Hence, edges with a higher number of placements are also allowed to have a higher variance. We again use a logarithmic scale because of the outlier. The figure reveals more details on the edges with lower variance, highlighted in medium green colors. Subfigure (d) shows the standard deviation of edge imbalances. Because we used imbalances of unit mass samples, the values are already normalized. The path to the Lactobacillus clade is again clearly visible, indicating that the placement mass in this clade has a high variance across samples. Note that imbalances can be negative; thus, the Index of Dispersion is not applicable to them. (TIFF) [file pone.0217050.s007.tiff]

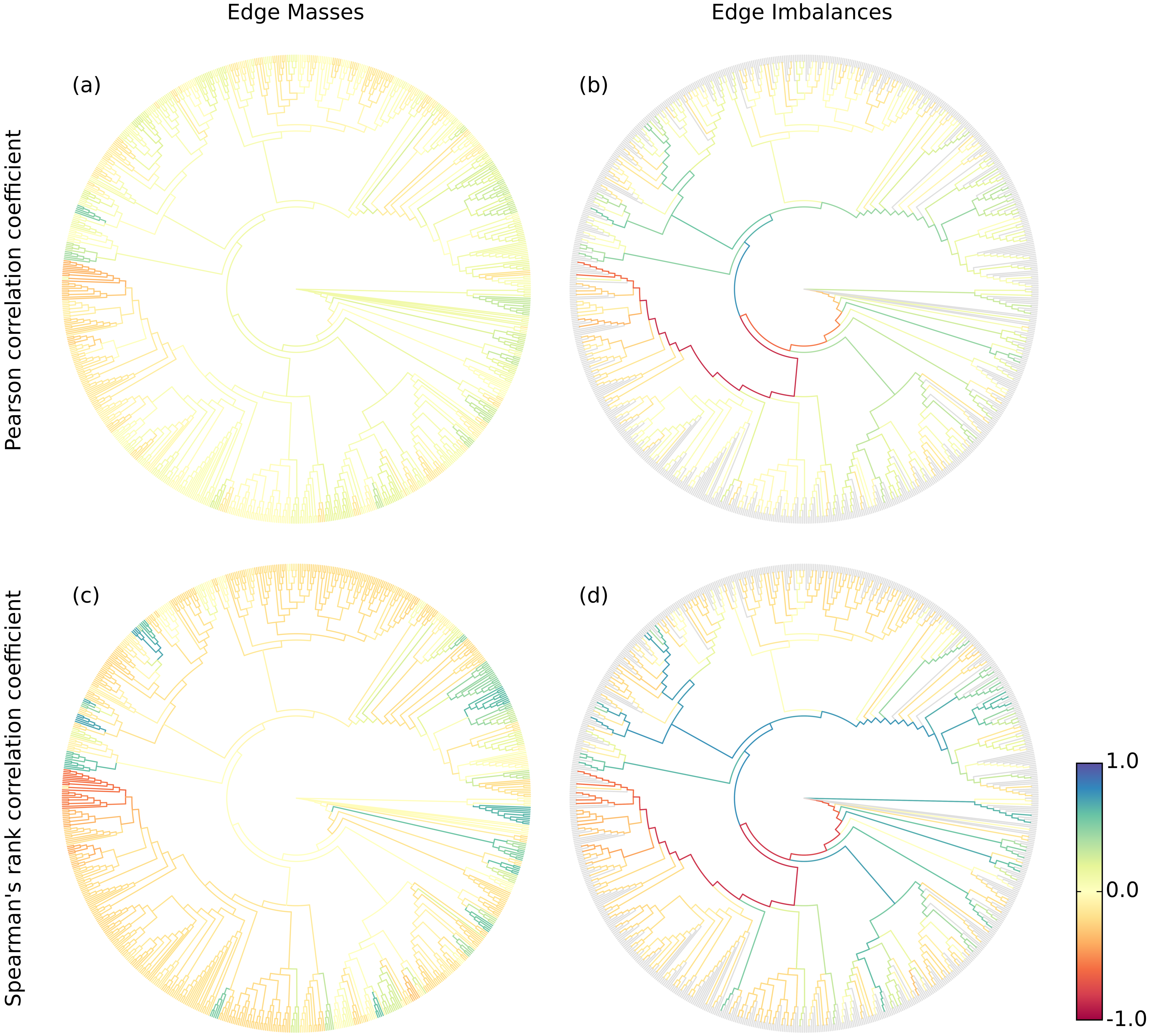

Supplement: S3 Fig — We again use the BV dataset, and show the correlation of edge masses and imbalances with the Nugent score. The Nugent score measures the severeness of Bacterial Vaginosis, and ranges from 0 for healthy subjects to 10 for heavily affected patients. Subfigures (a) and (b) use the Pearson Correlation Coefficient, that is, they show the linear correlation with the meta-data feature, while subfigures (c) and (d) use Spearman’s Rank Correlation Coefficient and thus show monotonic correlations. Subfigure (d) is identical to Fig 4(b) of the main text. All subfigures show red edges or red paths at the Lactobacillus clade. This indicates that presence of placements in this clade is anti-correlated with the Nugent score, which is consistent with the findings of [18] and [29]. In other words, presence of Lactobacillus correlates with a healthy vaginal microbiome. On the other hand, blue and green edges, which indicate positive correlation, are indicative of edges that correlate to Bacterial Vaginosis. The extent of correlation is larger for Spearman’s Coefficient, indicating that the correlation is monotonic, but not strictly linear. (TIFF) [file pone.0217050.s008.tiff]

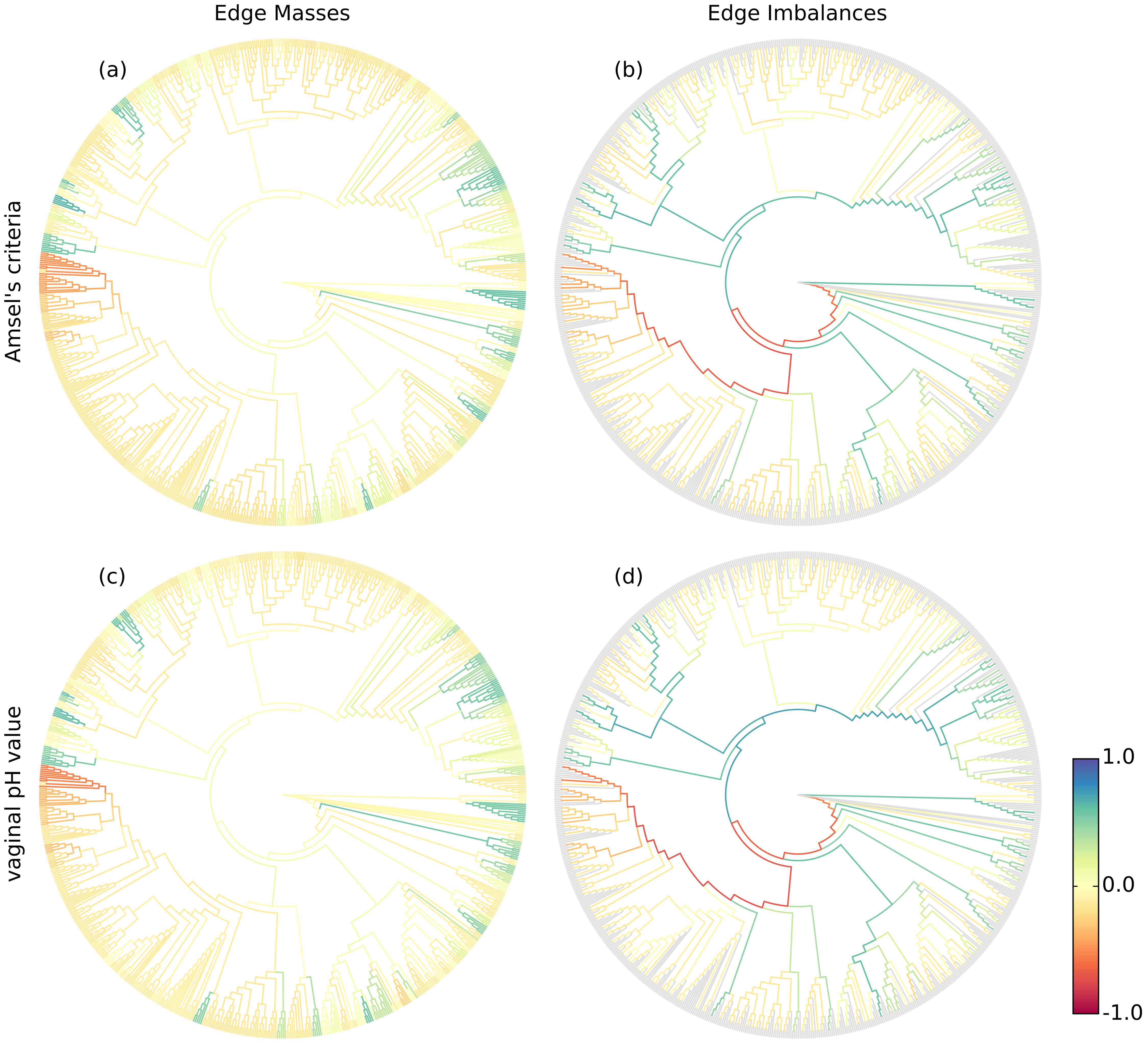

Supplement: S4 Fig — Here, we use additional meta-data features of the BV dataset to show that Edge Correlation yields consistent results with existing methods. In particular, we caltucated Spearman’s Coefficient with Amsel’s criteria [98] in subfigures (a) and (b), as well as with the vaginal pH value in subfigures (c) and (d). Both features were also used in [18] as indicators of Bacterial Vaginosis. The figures are almost identical to the ones shown in S3 Fig; that is, they yield results that are consistent with the previously used Nugent score, as well as consistent with existing methods. (TIFF) [file pone.0217050.s009.tiff]

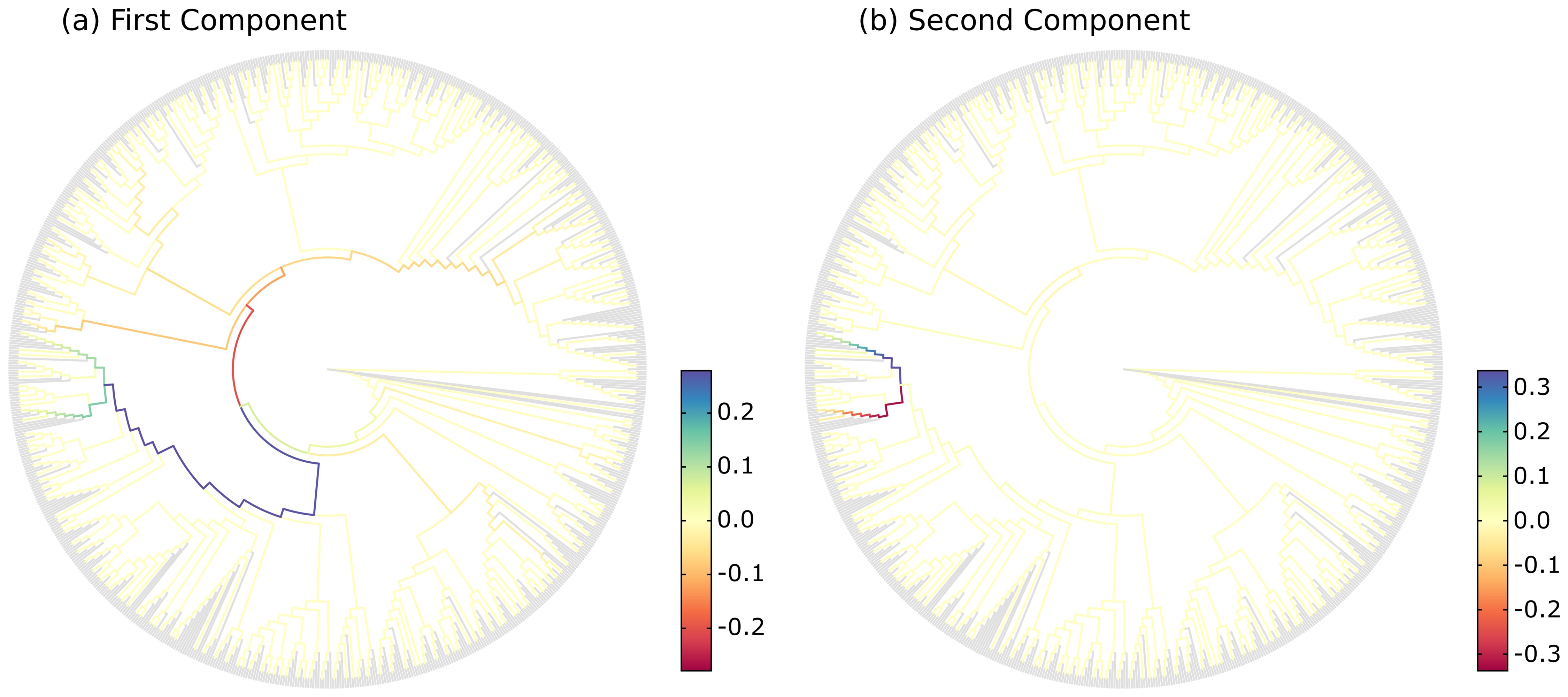

Supplement: S5 Fig — Subfigures (a) and (b) are recalculations of Figures 4 and 5 of [29], respectively. However, we show them here in our coloring scheme in order to facilitate comparison with other figures. The original publication instead uses two colors for a positive and a negative sign of the principal components, and branch width to show their magnitude. Note that the actual sign is arbitrary, as it is derived from principal components. The figure shows the first two Edge PCA components, visualized on the reference tree. This form of visualization is useful to interpret results such as the Edge PCA projection plot as shown in Fig 8(e) of the main text. It reveals which edges are mainly responsible for separating the samples into the PCA dimensions. Here, the first principal component in (a) indicates that the main PCA axis separates samples based on the presence of placements in the Lactobacillus clade, which is what the blue and green path leads to. The second component in (b) then further distinguishes between two species in this clade, namely Lactobacillus iners and Lactobacillus crispatus. (TIFF) [file pone.0217050.s010.tiff]

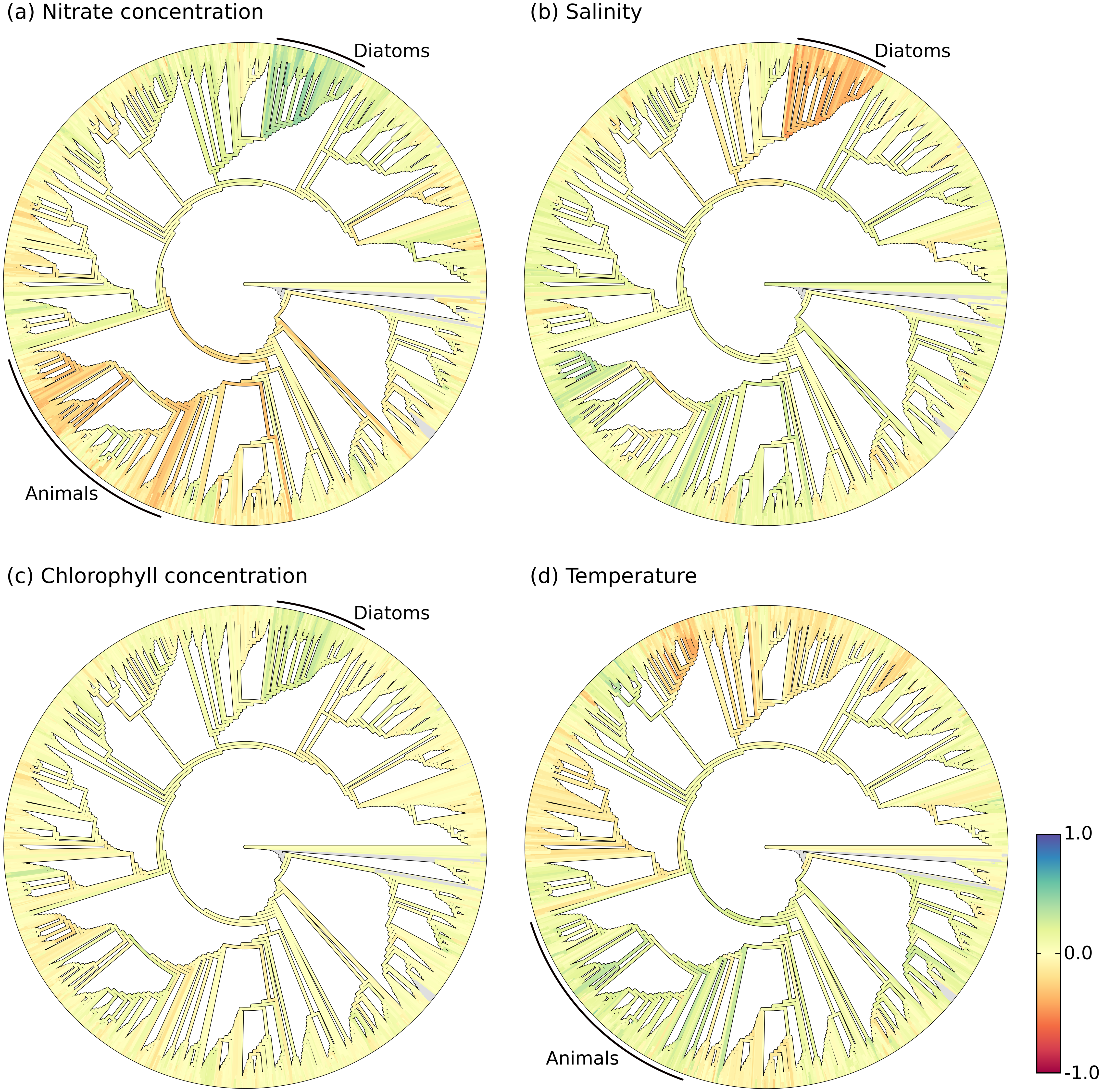

Supplement: S6 Fig — The figure shows the correlation of Tara Oceans sequence placements with (a) the nitrate, (b) the salinity, (c) the chlorophyll, and (d) the temperature sensor data of each sample. The sensor values range from −2.2 to 33.1 μmol/l (nitrate), from 33.2 to 40.2 psu (salt), from −0.02 to 1.55 mg/m3 (chlorophyll), and from −0.8 to 30.5°C (temperature), respectively. The negative nitrate and chlorophyll concentrations are values below the detection limit of the measurement method (pers. comm. with L. Guidi), and hence simply denote low concentrations. We used Spearman’s Rank Correlation Coefficient, and examine two exemplary clades, namely the Animals and the Diatoms. Diatoms are mainly photosynthetic, and thus depend on nitrates as key nutrients, which is clearly visible by the high correlation of the clade in (a). Furthermore, the diatoms exhibit positive correlation with the chlorophyll concentration (c), which again is indicative of their photosynthetic behavior. On the other hand, they show a high anti-correlation with the salt content (b). Salinity is a strong environmental factor which heavily affects community structures and species abundances [99], particularly diatoms [100]. The correlations of the animal clade are less pronounced. They exhibit a negative correlation with nitrate (a), as well as an increase in absolute abundance with higher temperatures (d). While these findings are not surprising, they show that the method is able to find meaningful relationships in the data. (TIFF) [file pone.0217050.s011.tiff]

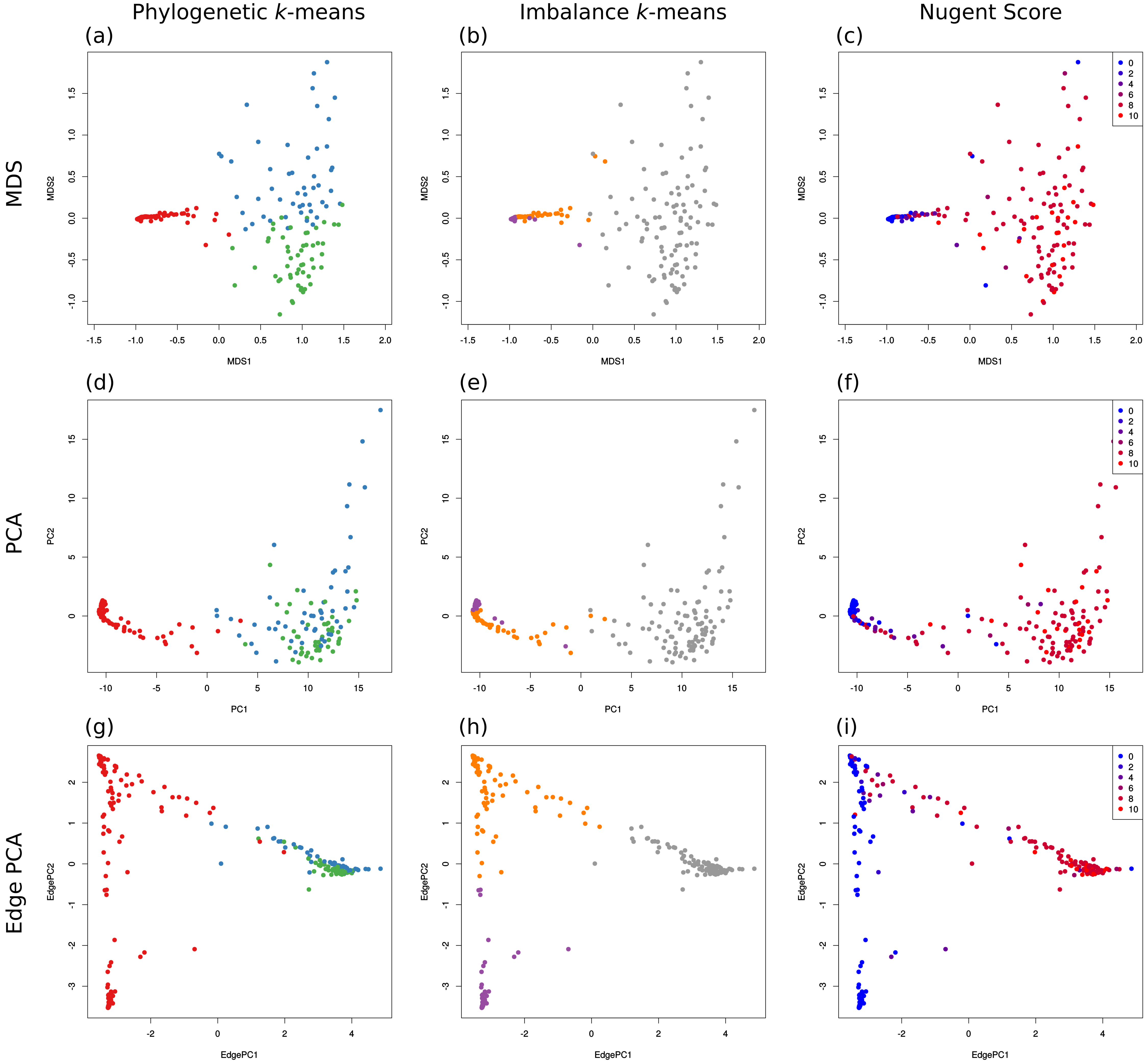

Supplement: S7 Fig — Here, we show and compare the dimensionality reduction methods MDS, PCA, and Edge PCA (one per row). MDS and PCA were calculated on the pairwise KR distance matrix of the BV dataset, Edge PCA was calculated using the placements on the re-inferred RT of the original publication [18]. The plots are colored by the cluster assignments as found by our k-means variants (first two columns), and by the Nugent score of the samples (last column). The Nugent score is included to allow comparison of the health status of patients with the clustering results. (a), (d) and (h) are identical to Fig 8(c), 8(d) and 8(e) of the main text, respectively. (f) and (i) are recalculations of Figures 4 and 3 of [101], respectively. This figure reveals additional details about how the k-means method works, that is, which samples are assigned to the same cluster. For example, the purple cluster found by Imbalance k-means forms a dense cluster of close-by samples on the left in (b) and (e), which is in accordance with the short branch lengths of this cluster as shown in Fig 8(b) of the main text. (TIFF) [file pone.0217050.s012.tiff]

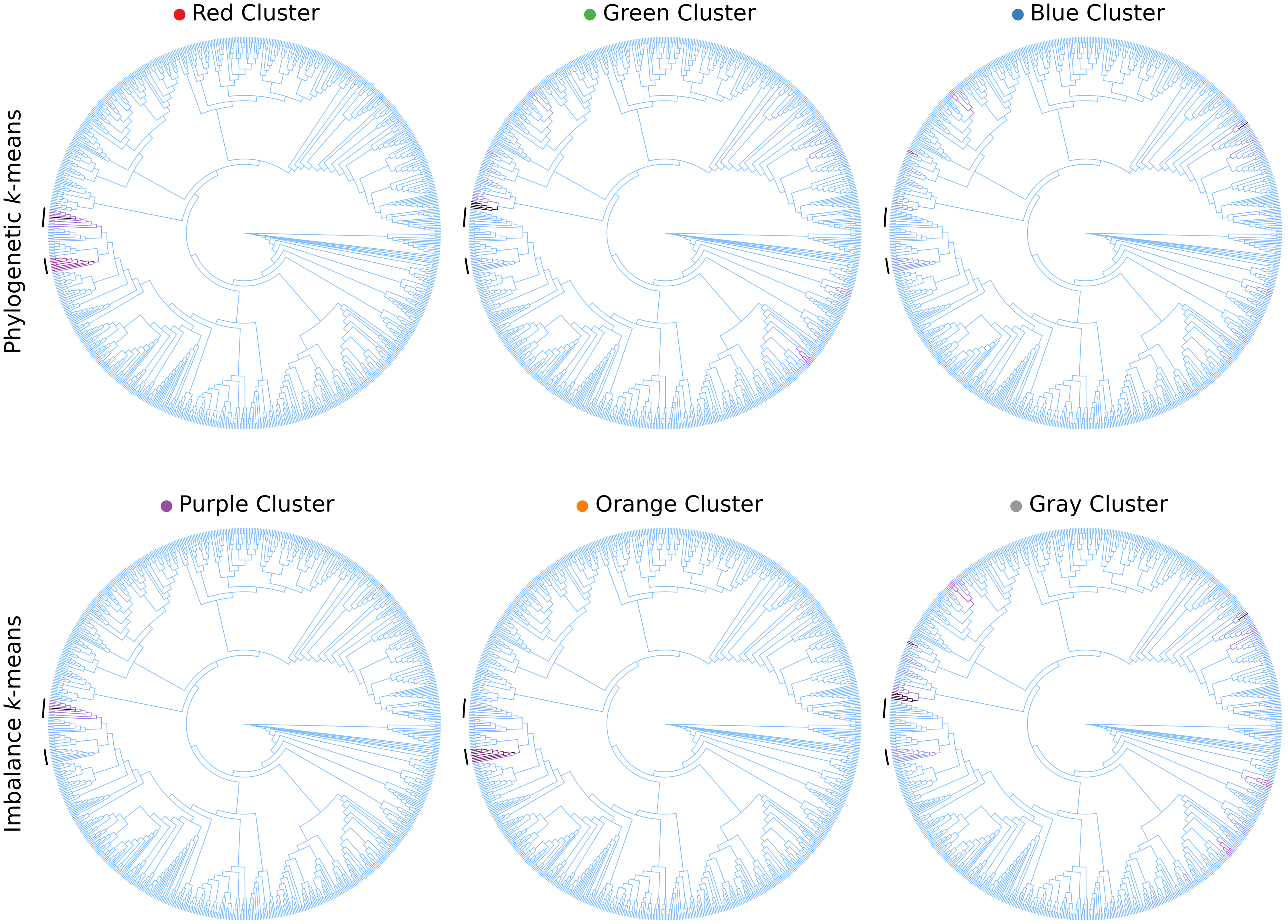

Supplement: S8 Fig — Here we show the cluster centroids as found by our k-means variants using the BV dataset, visualized on the reference tree via color coding. The cluster assignments are the same as in Fig 8 of the main text; the first row show the three clusters found by Phylogenetic k-means, the second row the clusters found by Imbalance k-means. Each tree represents one centroid around which the samples were clustered, that is, it shows the combined masses of the samples that were assigned to that cluster. The edges are colored relative to each other, using a linear scaling of light blue (no mass), purple (half of the maximal mass) and black (maximal mass). As explained in the main text, the samples can be split into three groups: The diseased subjects, which have placement mass in various parts of the tree, as well as two groups of healthy subjects, with placement mass in one of two Lactobacillus clades (marked with black arcs on the left of the trees). This grouping is also clearly visible in these trees. The red cluster for example represents all healthy subjects, and thus most of its mass is located in the two Lactobacillus clades. The purple and orange clusters on the other hand show a difference in placement mass between those clades. Furthermore, the placement mass of the gray cluster is mostly a combination of the masses of the green and blue cluster, all of which represent diseased subjects. These observations are in accordance with previous findings as explained in the main text. (TIFF) [file pone.0217050.s013.tiff]

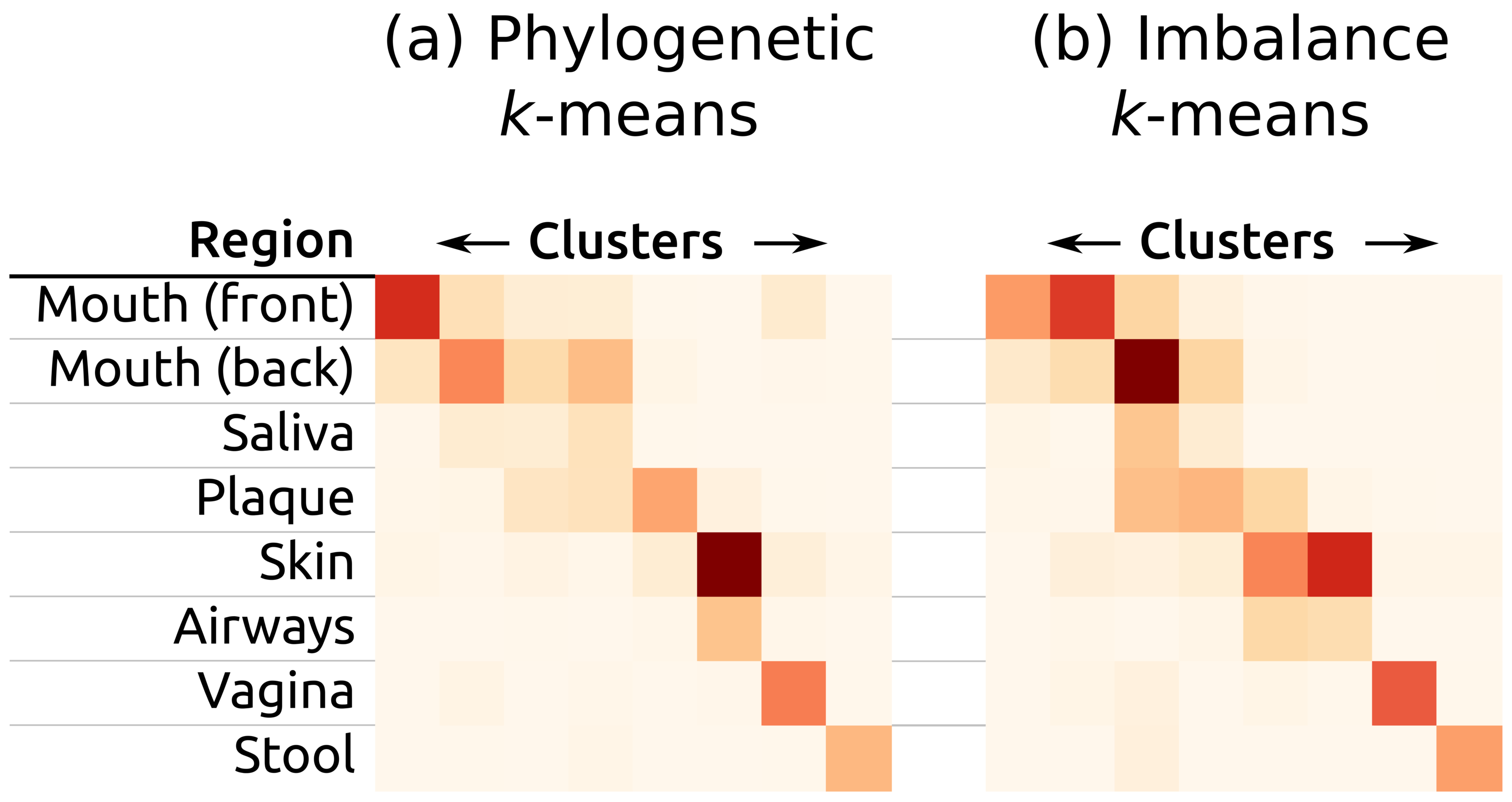

Supplement: S9 Fig — k is set to 8, instead of k ≔ 18 as in the main text, based on a coarse aggregation of the original body site labels as shown in S1 Table. See Fig 8 for the cluster assignment where k is set to the original number of labels; there, we also list how the labels were aggregated. Each row represents a body site; each column one of the 8 clusters. The color values indicate how many samples of a body site were assigned to each cluster. Some of the body sites can be clearly separated, while particularly the samples from the oral region are distributed over different clusters. This might be due to homogeneity of the oral samples. (TIFF) [file pone.0217050.s014.tiff]

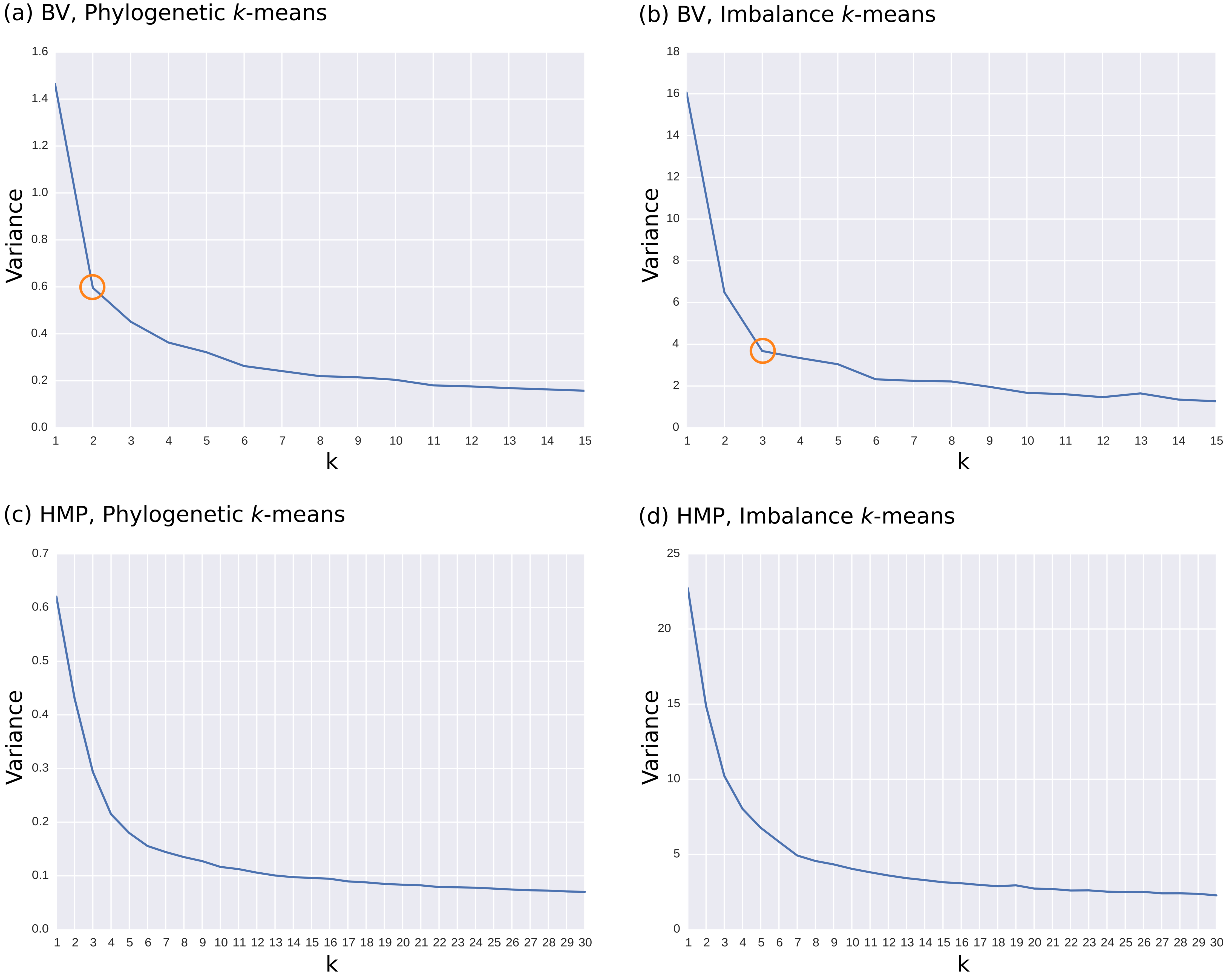

Supplement: S10 Fig — The figures show the cluster variance, that is, the average squared distance of the samples to their assigned cluster centroids, for different values of k. The first row are clusterings of the BV dataset, the second row of the HMP dataset. They were clustered using Phylogenetic k-means (first column), and Imbalance k-means (second column), respectively. Accordingly, (a) and (c) use the KR distance, while (b) and (d) use the euclidean distance to measure the variance. These plots can be used for the Elbow method in order to find the appropriate number of clusters in a dataset [79]. Low values of k induce a high variance, because many samples exhibit a large distance from their assigned centroid. On the other hand, at a given point, higher values of k only yield a marginal gain by further splitting clusters. Thus, if the data has a natural number of clusters, the corresponding k produces an angle in the plot, called the “elbow”. For example, (a) and (b) exhibit the elbow at k ≔ 2 and 3, respectively, which are marked with orange circles. These values are consistent with previous findings, for instance, Fig 8: There, Phylogenetic k-means splits the samples into a distinct red cluster and the nearby green and blue clusters, while Imbalance k-means yields three separate clusters in purple, orange, and gray. For the HMP dataset, the elbow is less pronounced. We suspect that this is due to the broad reference tree not being able to adequately resolve fine-grained differences between samples, see S1 Text for details. Likely candidates for k are 4–6 for (c) and around 7 for (d). These values are consistent with the number of coherent “blocks” of clusters, which can be observed in Fig 9. Clearer results for this dataset might be obtained with other methods for finding “good” values for k, although we did not test them here. (TIFF) [file pone.0217050.s015.tiff]

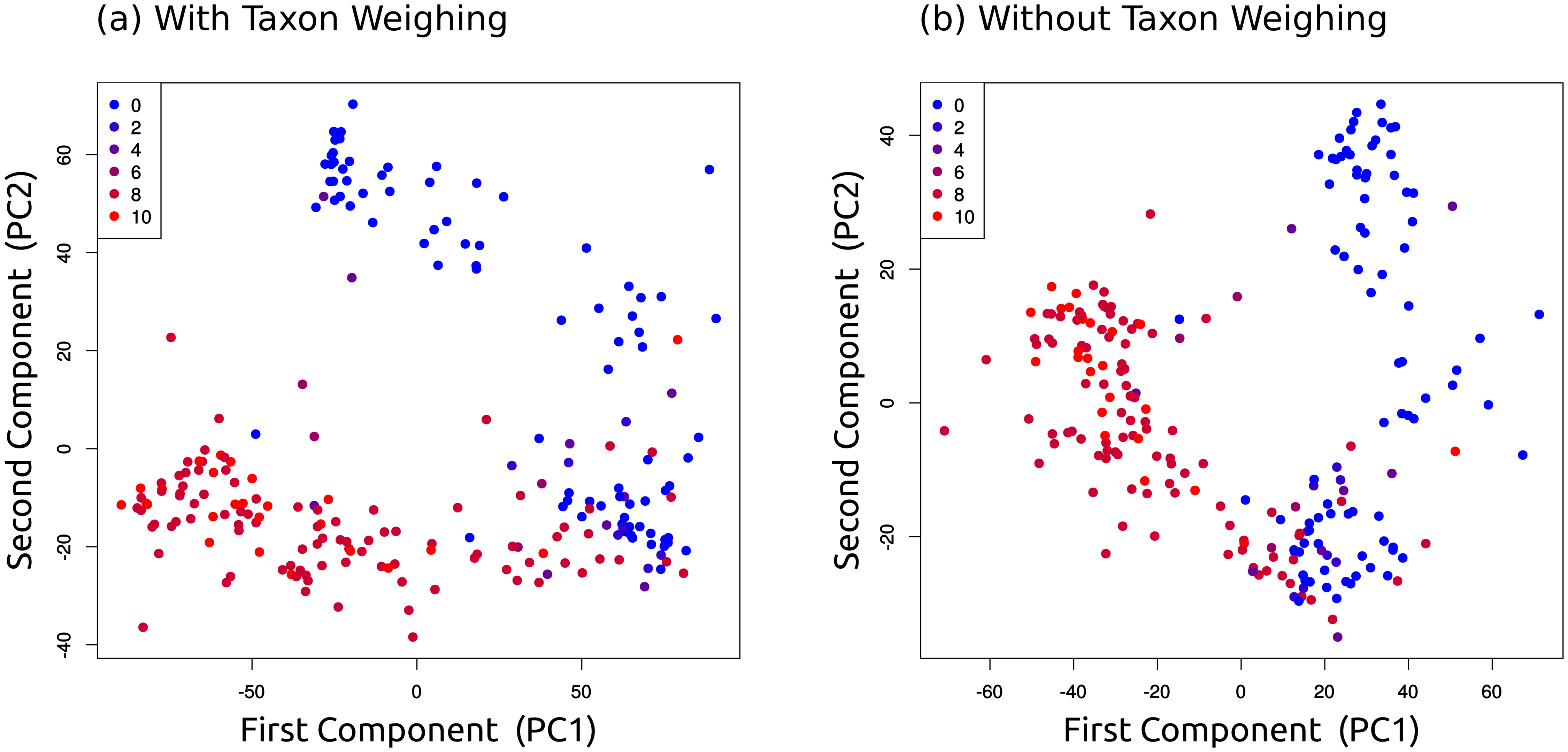

Supplement: S11 Fig — The plots show the first two principal components of a PCA on the per-edge balances, calculated on placements of the data on reference tree of the BV dataset. That is, for each edge of the tree, we calculated the balance (log-ratio of geometric means) of the placement masses of the BV samples between the two sides of the tree induced by the edge. (a) shows the result when using taxon weighting [30] in the balances calculation, while (b) shows the result without taxon weighting. Each item represents a sample, colored by its Nugent score (0 means healthy, 10 means severe illness); the Nugent score had no influence on the PCA calculations. Both plots separate the healthy from the sick patients. In contrast to Edge PCA, the first component of (a) does not fully distinguish between the healthy (blue) and diseased (red) samples. For some reason, the component only takes Lactobacillus iners into account, while mostly ignoring Lactobacillus crispatus. This can be seen in S12(a) Fig, which shows the eigenvectors of this component visualized on the reference tree. There, the path leading to the Lactobacillus clade does not include the branches of Lactobacillus crispatus, which is marked with a black arc. Including the second component however, which distinguishes between the two types of Lactobacillus, as shown in S12(b) Fig, yields a clear separation of the samples. Subfigure (b) exhibits closer similarities to the Edge PCA plot shown in S7(i) Fig, in that the first component separates healthy from sick, and the second component further splits the healthy individuals apart. The edges that are responsible for these splits can be seen in more detail in S12 Fig, where we visualize the first two principal components of the balances without taxon weighting on the tree. (TIFF) [file pone.0217050.s016.tiff]

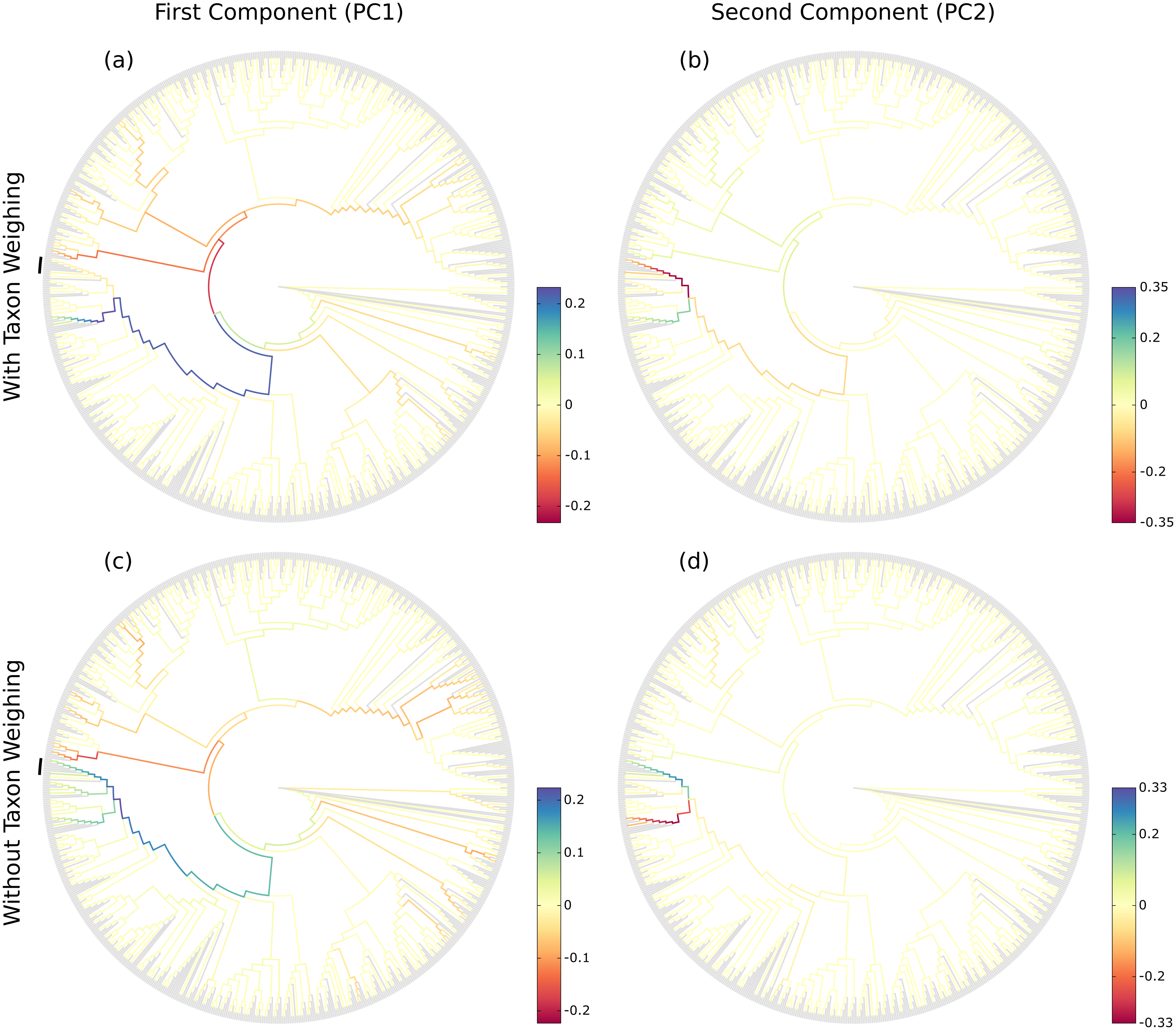

Supplement: S12 Fig — The figure shows the eigenvectors of the first two principal components of PCA on the per-edge balances with and without taxon weighting, visualized on the reference tree of the BV dataset. See S11 Fig for details on the balances calculation. The visualization of the components on the reference tree is analogous to the Edge PCA tree visualization as for example shown in S5 Fig. As the data that is considered in the PCA corresponds to the edges of the tree, the resulting eigenvectors can be mapped back onto the tree, which is shown here. Each edge is colored according to the corresponding value of the first principal component in (a) and (c), and the second principal component in (b) and (d), respectively. The figure hence indicates how the axes in S11 Fig can be interpreted: The first component leads to the Lactobacillus clade, while the second one splits this clade into Lactobacillus iners and Lactobacillus crispatus. Hence, the results obtained from this analysis are consistent with our previous findings, c. f., S5 Fig and [18]. In (a) and (c), we marked the Lactobacillus crispatus clade with a black arc at the left of the tree. (TIFF) [file pone.0217050.s017.tiff]

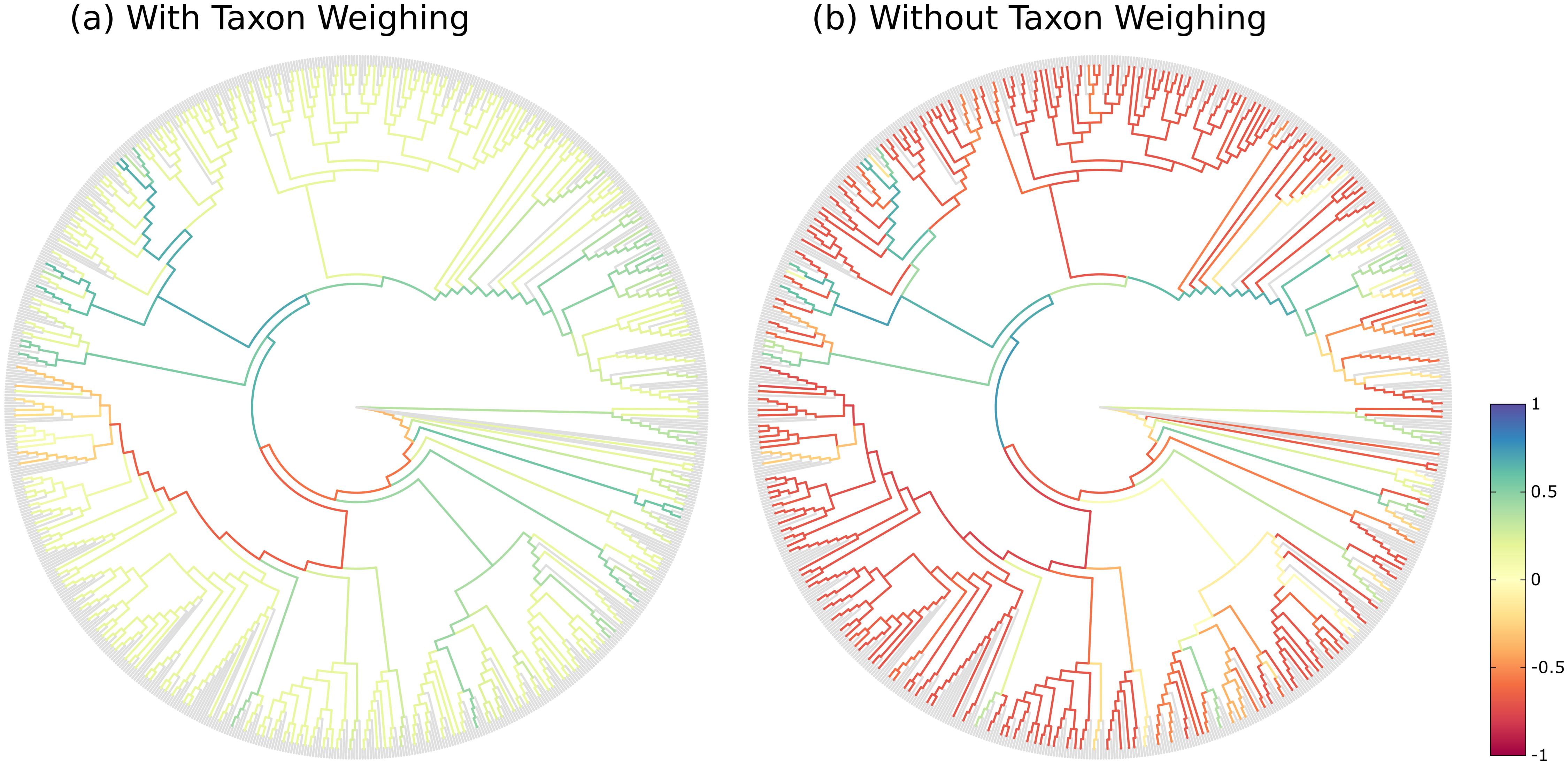

Supplement: S13 Fig — The Edge Correlation method as presented in the main text, and for example shown in S3 Fig, can also be conducted using balances (instead of masses or imbalances). We here show Edge Correlation using Spearman’s Rank Correlation Coefficient, calculated on the per-edge balances and the Nugent score, based on the placement of the BV dataset. That is, for each edge of the tree, we calculated the balance (log-ratio of geometric means) of the placement masses of the BV samples between the two sides of the tree induced by the edge. Then, we calculated the correlation with the Nugent score of each sample, and visualized it on the tree. (a) shows the result when using taxon weighting [30] in the balances calculation, while (b) shows the result without taxon weighting. With taxon weighting in (a), the result is similar to the correlation with imbalances shown in S3(d) Fig: An anti-correlation with the Lactobacillus clade is again visible (less placement mass in this clade means higher Nugent score, that is, indicates a more severe illness), while several other clades exhibit a positive correlation with Nugent score. The most striking difference to the previous Edge Correlation trees in S3 Fig is the majority of spuriously anti-correlated (red) edges in the tree without taxon weighting in (b). As mentioned in the main text, this is due to the insensitivity of the geometric mean to the presence of singular large values: If most values are small, so will be their geometric mean, even if a few very large values are also present. As can be seen in S1 and S2 Figs, the clades that exhibit a high anti-correlation here (red) have low placement mass with a low variance. Hence, the geometric mean of the masses in these clades is consistently low across samples, which means that the numerator of the log-ratio in the balances computation has little effect on the correlation. This implies that the denominator, which represents the rest of the tree, drives the anti-correlations seen here. W [file pone.0217050.s018.tiff]

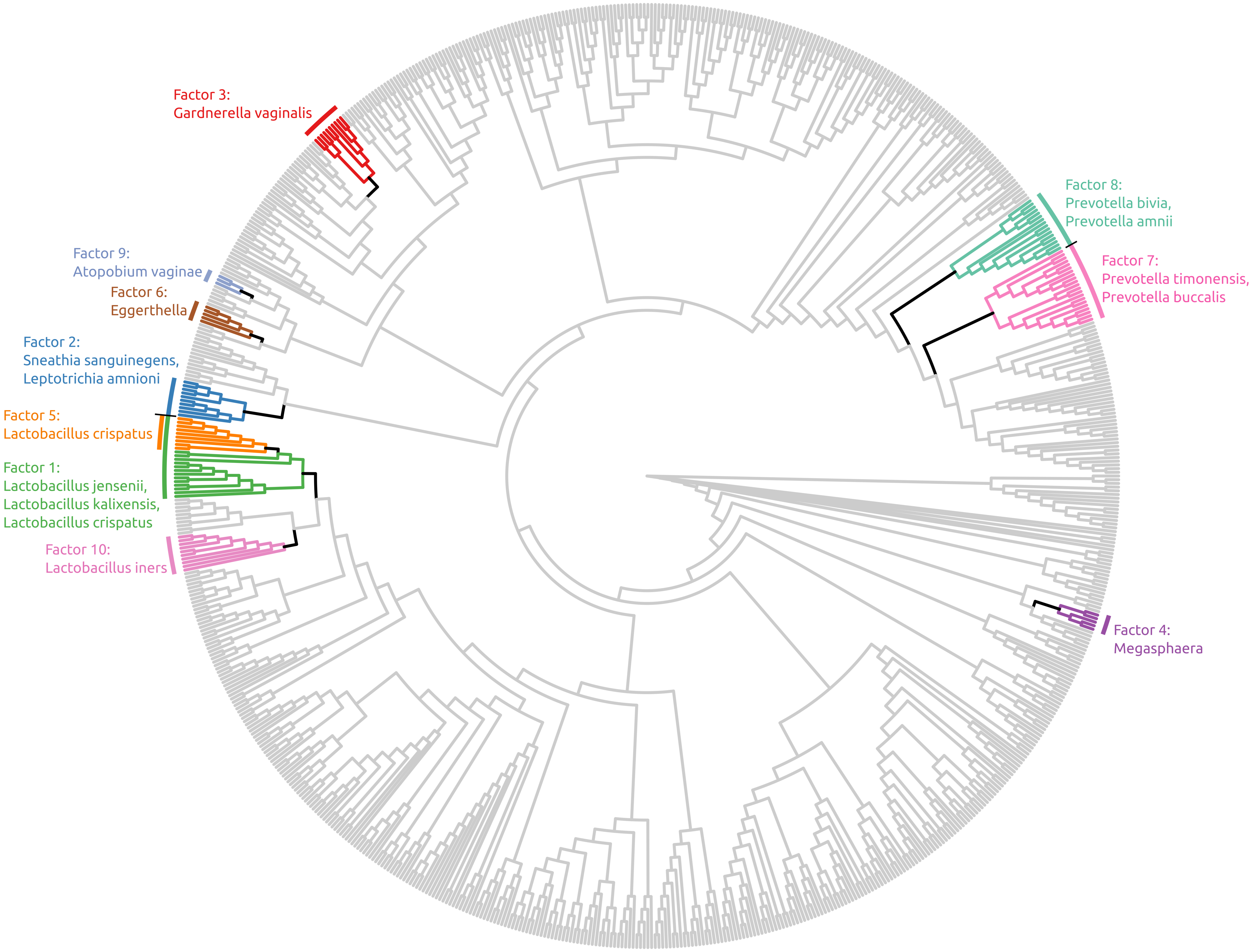

Supplement: S14 Fig — Here, we show the first ten factors found by Placement-Factorization without taxon weighting on the BV dataset. The black edges are the winning edges of each iteration, which split the tree into several subtrees. For simplicity, we only colored the clades leading away from the (arbitrarily placed) root, while leaving the paraphyletic “remainder” clade in gray. Note that factor 5 is nested in factor 1, that is, it further splits the branches within the first factor, thus separating Lactobacillus crispatus from Lactobacillus jensenii and Lactobacillus kalixensis. See S3 Table for a comparison of the clades separated by each factor to the factors found by the original Phylofactorization. Furthermore, see S16(b) Fig for an ordination of the first two factors, showing how these factors separate the samples in the dataset. The clades found here are consistent with the findings of the original study of the dataset [18]: Healthy women without BV exhibit high abundances of Lactobacillus, while women affected by BV have a more diverse vaginal microbiome, containing multiple different bacterial taxa. Hence, the first factor represents the most prominent split of the data into healthy vs. diseased, based on the presence of Lactobacillus. Differences within the healthy samples are then further distinguished in factors five and ten, which further split parts of the Lactobacillus clade. The remaining factors split away clades that further separate the diseased samples from each other, based on several bacterial taxa. All clades that are found by these factors where shown in the original study to be associated with BV [18], meaning that Placement-Factorization on this dataset yields results that are consistent with previous analyses. (TIFF) [file pone.0217050.s019.tiff]

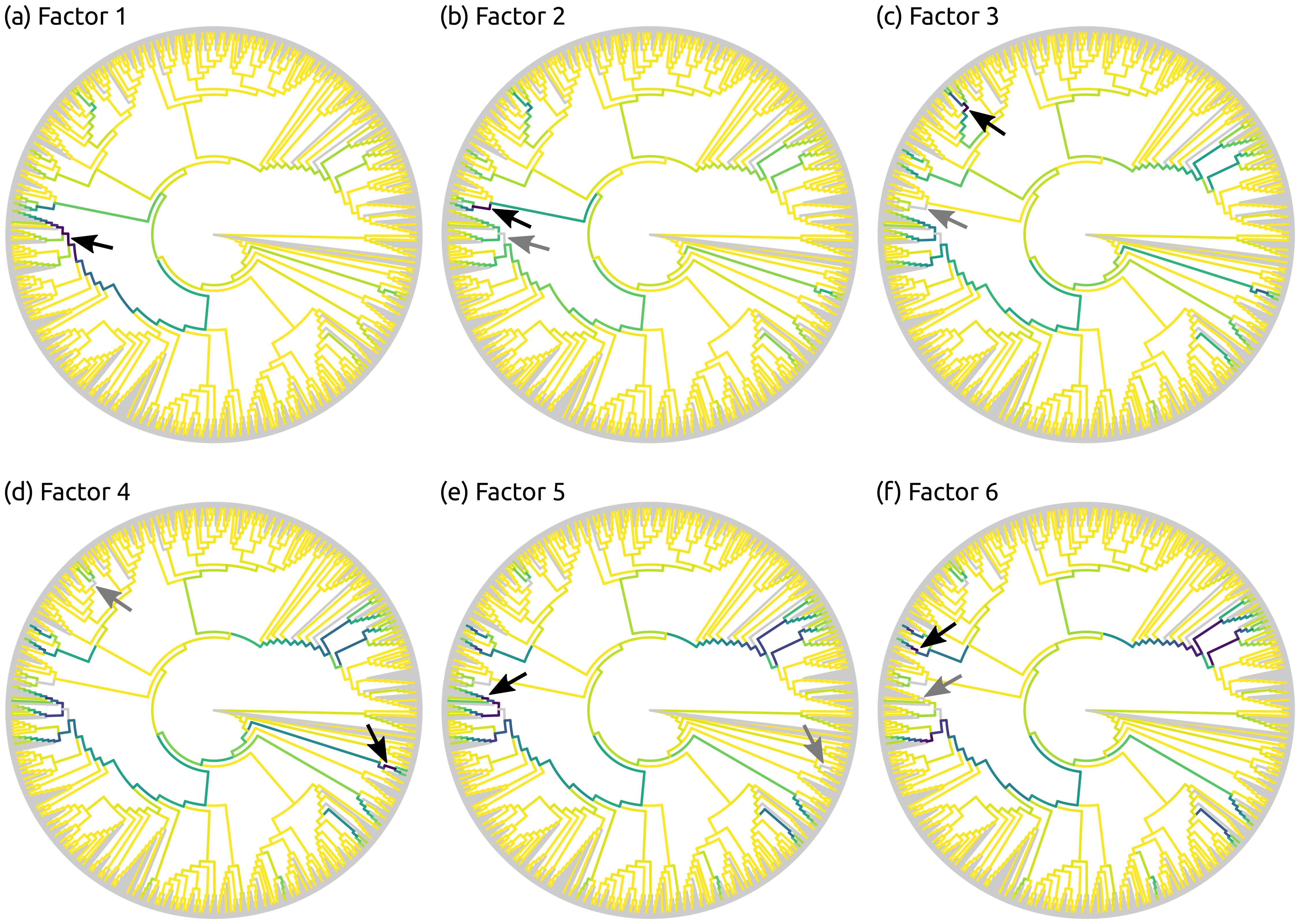

Supplement: S15 Fig — In each iteration of Phylofactorization and Placement-Factorization, the objective function is evaluated for each edge of the tree (except for edges that were winning previous iterations). The figure visualizes the value of the objective function for the first six iterations of Placement-Factorization of the BV dataset, without taxon weighting. Darker edges represent higher values; the highest value of each iteration (the winning edge) is marked with a black arrow. Gray arrows further mark the winning edge of the respective previous iteration, which allows to examine the effect of “factoring out” an edge: Due to the nature of comparing the two sides induced by an edge, high values of the objective function usually propagate across several connected edges, e. g., the region of dark branches around the marked edge in (a). Once a factor has been split from the tree, the values for the whole path drop, which can be seen by comparing (a) and (b), where the region around the gray arrow has much lower values of the objective function. This behavior can consistently be observed in the other subfigures as well. The clades of the winning edges shown here can also be seen in S14 Fig, and are listed in S3 Table. See also Fig 10 for the according visualization with taxon weights. (TIFF) [file pone.0217050.s020.tiff]

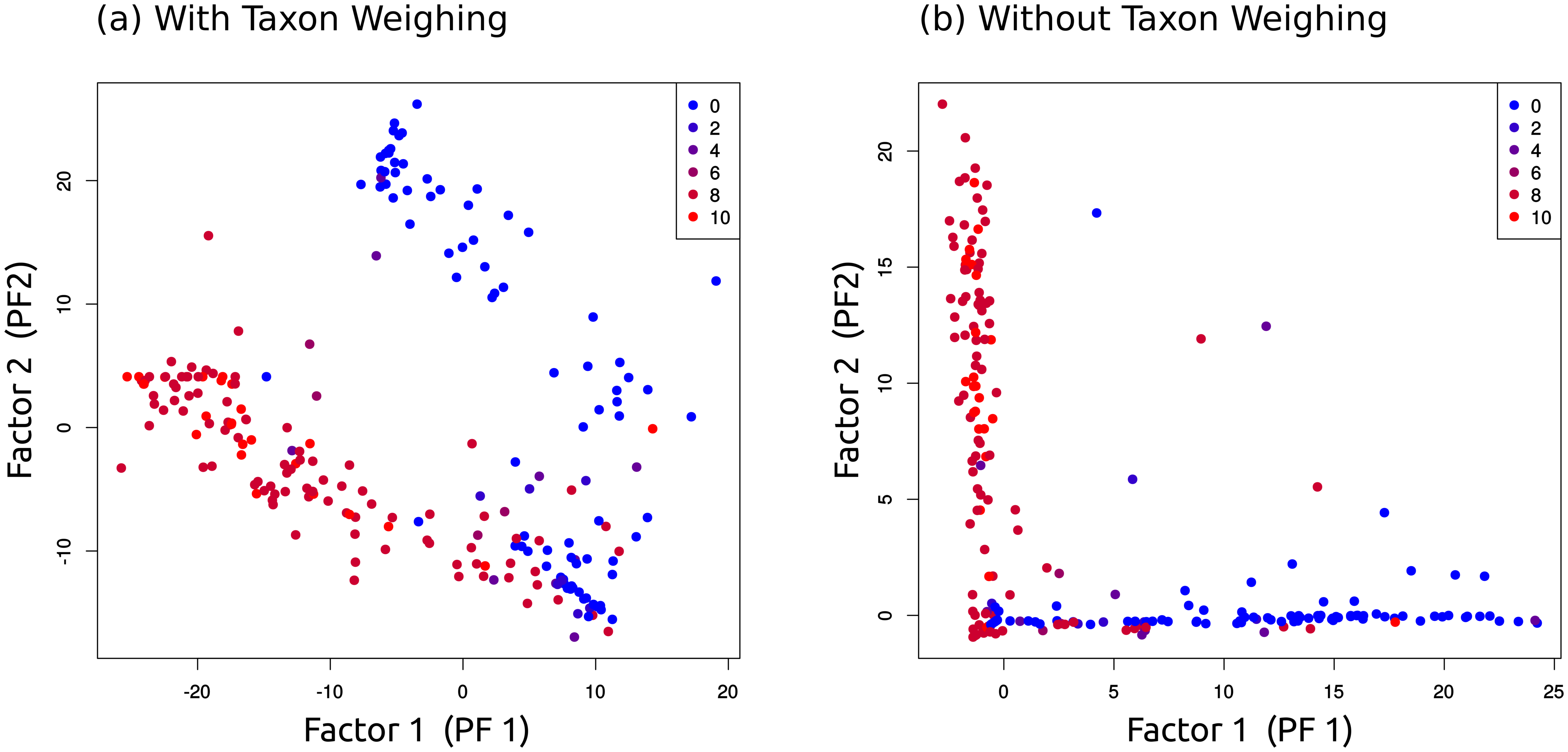

Supplement: S16 Fig — The figure shows ordination-visualization plots of the ILR coordinates of the first two factors found by Placement-Factorization of the BV dataset, (a) with and (b) without taxon weighting. That is, the axes correspond to the splits induced by the first two factors, while values along the axes are the balances of each sample calculated on the sets of edges of each split. Samples are again colored by their Nugent score, with 0 being the healthy patients, and 10 being the patients with severe BV. See Fig 10 for the (winning) edges that correspond to the axes in (a) (with taxon weighting), and see S14 Fig and S3 Table for the edges corresponding to the axes in (b) (without taxon weighting). This type of plot was suggested in [31] as an additional way of depicting how the factors separate samples according to meta-data features, see Figure 5(a) of [31]. Note that such visualizations can only reasonably visualize the first two or three factors, which is why they are now discontinued in the original Phylofactorization (pers. comm. with A. Washburne on 2019-01-16). For the BV dataset however, this suffices to show the major features. We also developed a way of visualizing further factors, as for example shown in S18 Fig, which alleviates this issue. On the one hand, Placement-Factorization with taxon weighting in (a) is highly similar to PCA on the balances as shown in S11(a) Fig, despite the fact that PCA does not take the meta-data into account. We suspect that is is due to the nature of the dataset, were the abundances in the Lactobacillus clade almost solely dictate the health status of each individual, and roughly half the samples belong to either the healthy or the sick group of patients. Hence, the Lactobacillus clade naturally is a major driver of differences between samples, and is thus identified by PCA as the most important component/axis. On the other hand, Placement-Factorization without taxon weighting in (b) yields an ordination that separates healthy from s [file pone.0217050.s021.tiff]

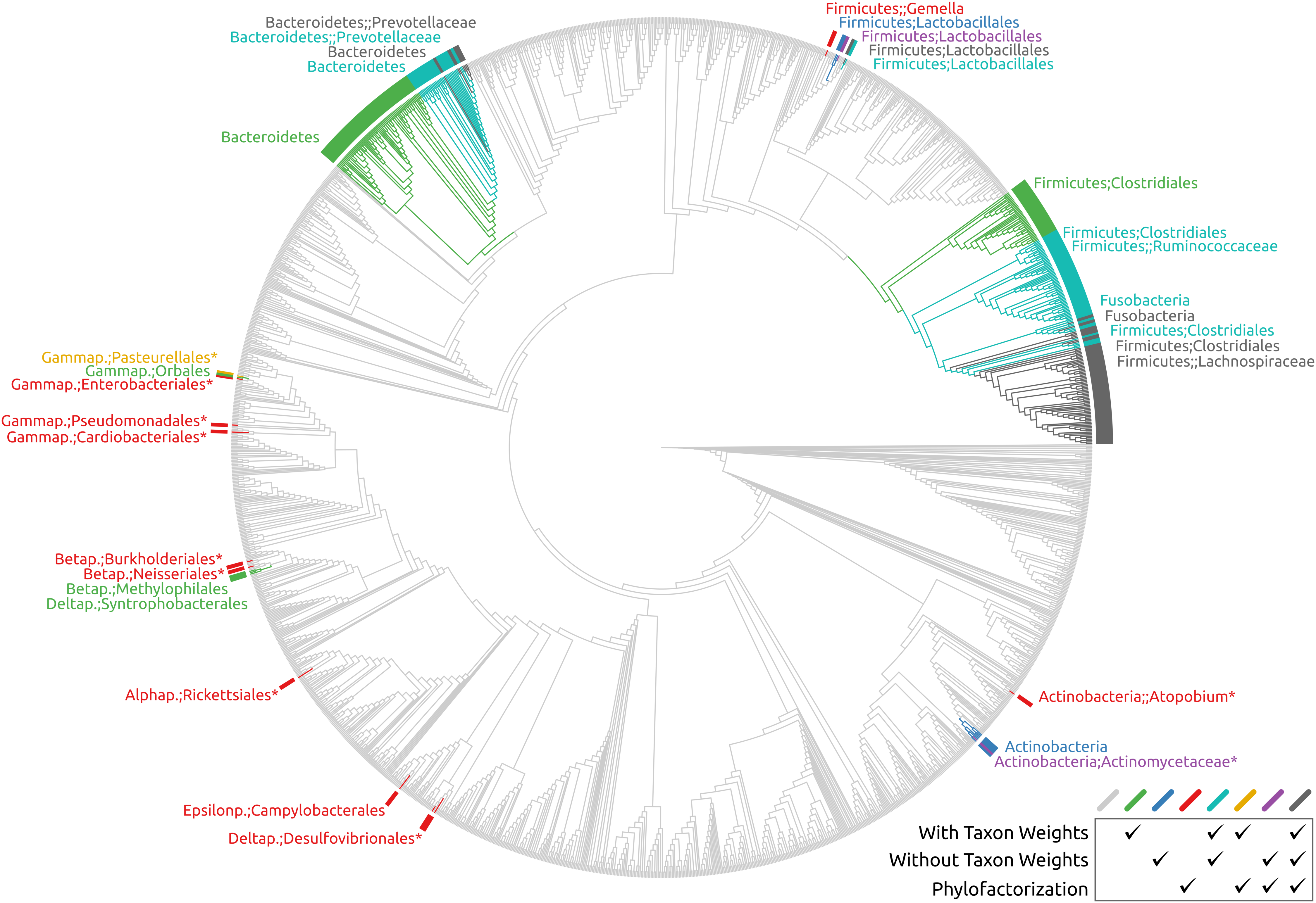

Supplement: S17 Fig — Here, we compare the first 10 factors found by Placement-Factorization with and without taxon weighting on an oral/fecal subset of the HMP dataset to the first 10 factors found by Phylofactorization on their oral/fecal test dataset [31, 104]. To this end, we mapped the taxa of the latter to the Silva taxonomy [105, 106] that was used for constructing the reference tree shown here [34]. The clades of the tree are colored so that green, blue, and red mark branches that only appear in one of the variants, cyan, yellow, and purple for branches that occurred in two variants, and dark gray for branches that were found by all three variants. For simplicity, we here neglect the order and nesting of factors. That is, if a branch is part of the non-root side of one of the first ten factors, it is colorized here. It is striking that the variant with taxon weights (green) finds larger clades than the other two. We already observed a similar behavior with the BV dataset. However, the values of the objective function indicate that the focus of the factor is in fact much smaller and more in agreement with the other two variants shown here. Again, this behavior is similar to the BV data with taxon weighting; see Fig 10 for details. Furthermore, the several small clades and single branches found by Phylofactorization (red) that are part of the Actinobacteria as well as the Alpha-, Beta-, Gamma-, and Deltaproteobacteria are actually all part of the first factor. They are marked with asterisks (*) here. Due to their OTU tree only having few Proteobacteria, these were monophyletic in their tree. They are polyphyletic here, as our tree has more reference taxa from that group. Most of the remaining factors found by Phylofactorization are part of the gray branches of the two large clades in the figure, which are the clades that were found by all three variants. Similarly, our variants found many nested factors (factors that further split a factor of a previous iteration): The two large cl [file pone.0217050.s022.tiff]

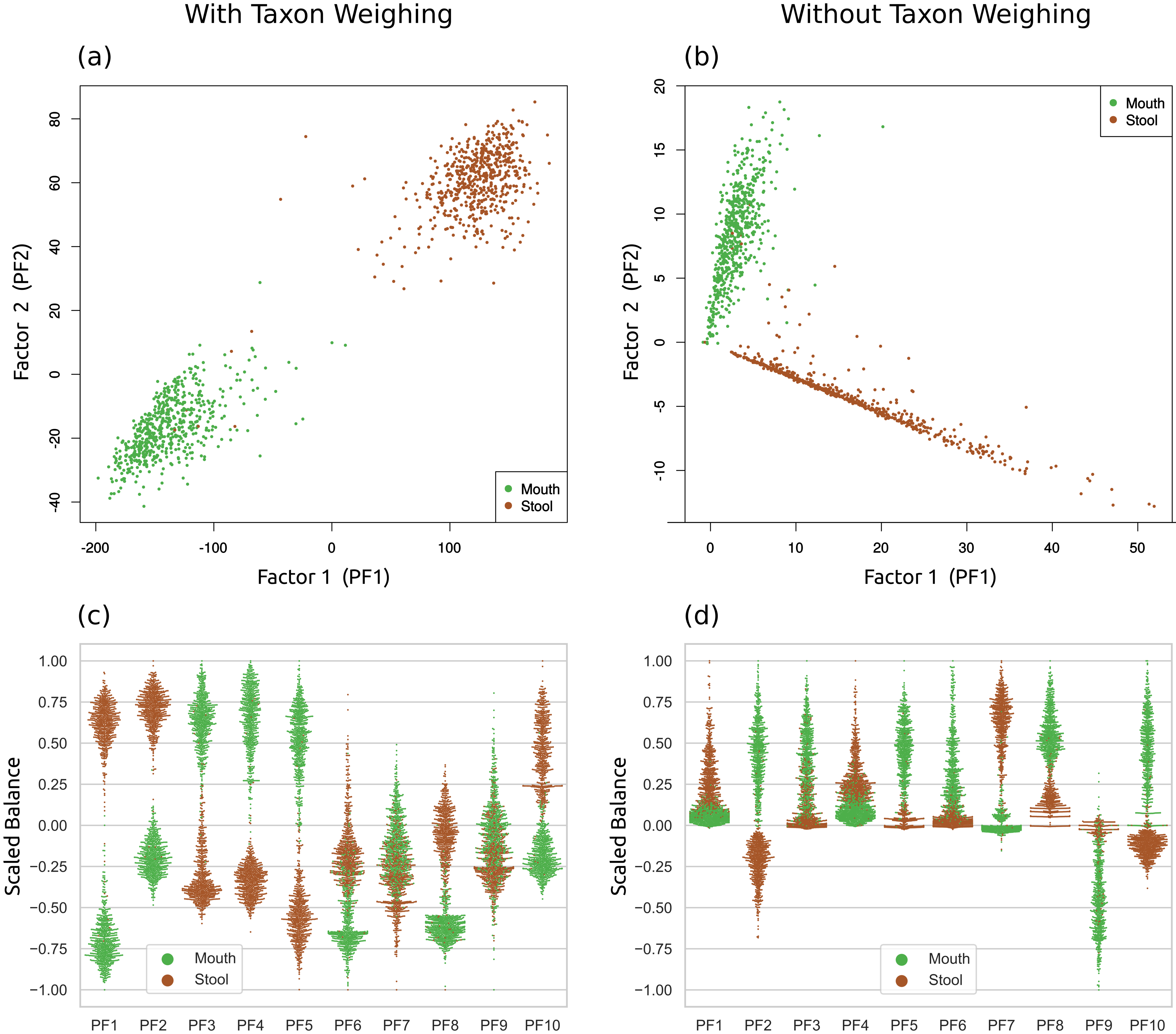

Supplement: S18 Fig — The figure shows the ordination-visualization of factors found by Placement-Factorization on our oral/fecal subset of the HMP dataset. In (a) and (b), we show the balances at the winning edges of the first two factors, colorized by the body site of each sample, with and without taxon weighting. In particular, (a) exhibits a clear separation of the two body sites, similar to Figure S3 of [31]. Moreover, in order to examine how well further factors of later iterations split the data, we here employ a visualization of phylofactors, which we call balance swarm plots, by plotting the balances of each factor individually. This type of per-factor visualization is similar to, e. g., Figure 4 of [92]. Subfigures (c) and (d) show the first ten factors (PF1–PF10), again with and without taxon weighting, respectively. These can be understood as multi-dimensional scatter plots, where each dimension is shown separately: Each column corresponds to a factor, with the vertical axis being the balances, and horizontal space within each column used to spread samples at nearby positions, revealing their distribution density. That is, the first two columns of (c) and (d) correspond to the scatter plots of (a) and (b), respectively. The balances were scaled to bring them into the [−1.0, 1.0] interval for better comparability across factors, while keeping the centering at 0. Subfigure (c) is identical to Fig 11(a) of the main text, and shows that almost all factors individually suffice to separate the data by body site; in (d), the separation is still present, but not as distinguished. (TIFF) [file pone.0217050.s023.tiff]

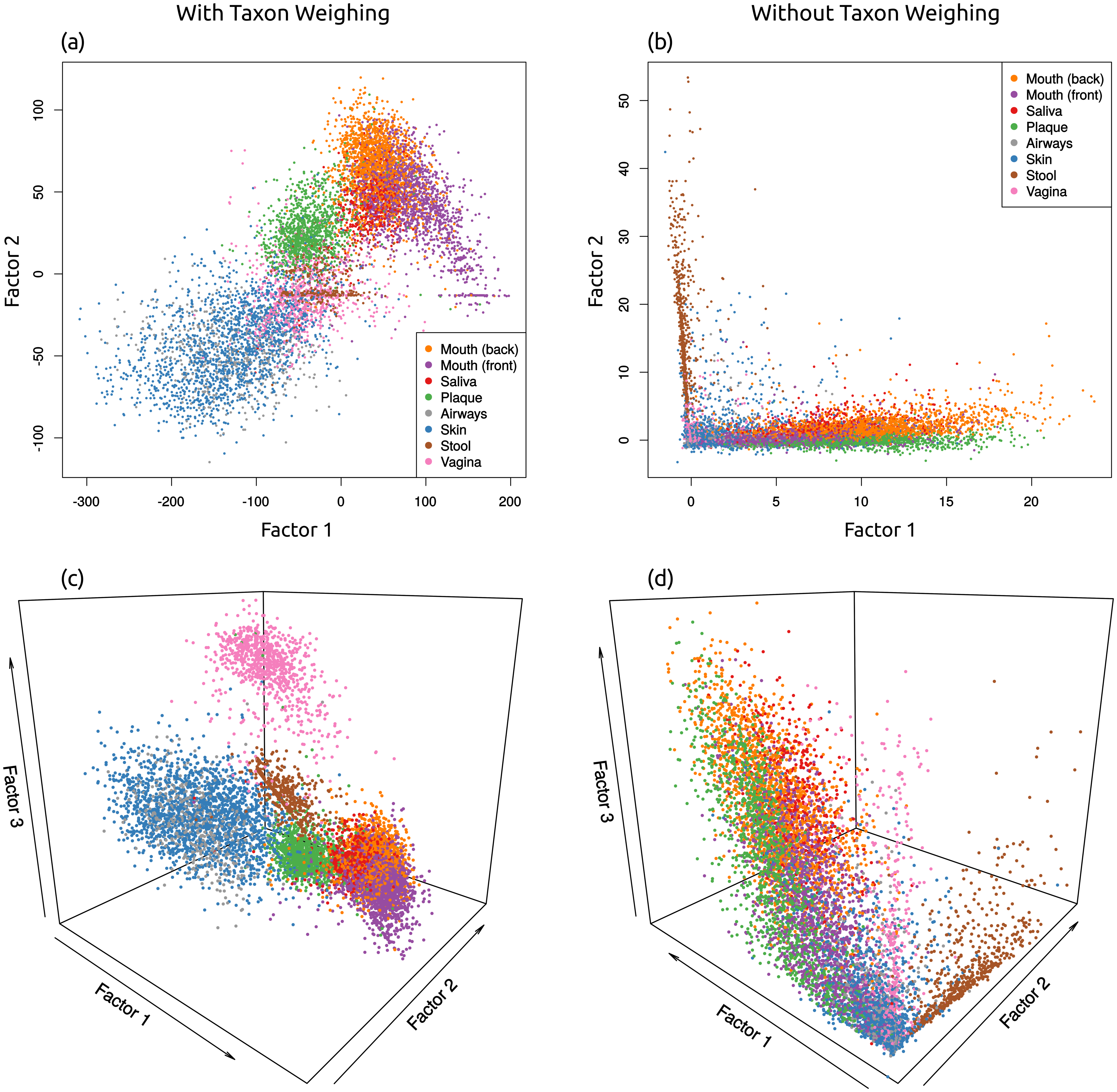

Supplement: S19 Fig — In S18 Fig, we use an oral/fecal subset of the HMP dataset [16, 17] to compare Placement-Factorization to a case study of the original Phylofactorization. Here, we instead used the whole HMP dataset, labeled by 8 body site regions as listed in S1 Table, to asses how well Placement-Factorization with a GLM objective function can separate samples based on their body site label. Again, we show the balances of the winning edges of the first two and three factors, respectively, with and without taxon weighting. As in S16 and S18 Figs before, the plots with taxon weighting form “clouds”, whereas the plots without form an “L”-shape. In all cases, a separation of the oral samples from the other regions is clearly visible. Noticeably, in (a), a part of the stool and mouth samples form a horizontal line, which indicates that the second factor does not distinguish between samples from those regions. It is striking that the samples from the vaginal region in (a) and (c) are not separated from the other samples until the third factor, which is visible as a pink cloud above the rest of the samples in (c). This again serves as a caveat that one needs to consider enough factors in order to get a complete understanding of the results. To this end, we furthermore show a more detailed version of these scatter plots for multiple factors/dimensions in S20 Fig. (TIFF) [file pone.0217050.s024.tiff]

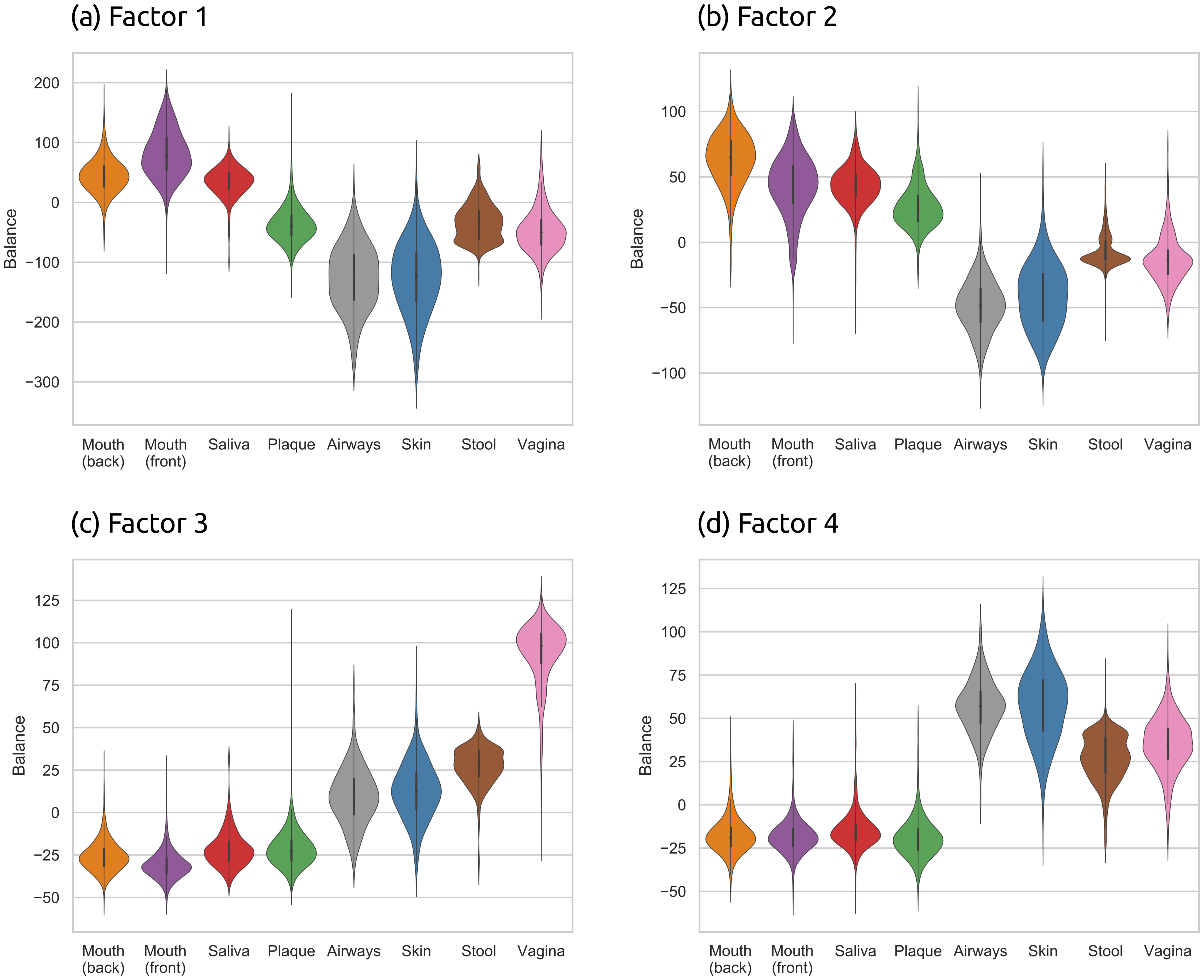

Supplement: S20 Fig — The balance swarm plots as shown in S18(c) and S18(d) Fig allow for a more detailed understanding of how each factor separates the samples. They can be colored by either continuous meta-data variables, similar to Figure 5(a) of [31], or a categorical variable with a limited number of categories, as shown in S18 Fig. However, for the eight body regions that we use for the HMP data, this type of visualization becomes hard to inspect visually. Hence, we here extend on the idea of balance swarm plots, and show the distribution of balances for each factor and for each body sites separately. That is, each subfigure here shows the balances of the winning edge of a factor, grouped by the categorical meta-data variable body site. In other words, each subfigure here represents a disentangled column of a balance swarm plot, where each body site is displayed by its own violin. The data shown here is again the result of Placement-Factorization with taxon weighting on the full HMP dataset, as shown and explained in S19 Fig. See there for the scatter plots that correspond to the first two and three factors shown here. Subfigure (a) is identical to Fig 11(b) of the main text, and included here for comparability. In all subfigures, the oral regions are separated from the other regions: For example, in (a), mouth and saliva samples exhibit balances above 0, in contrast to all other samples. In (b), (c), and (d), the plaque samples join the other oral samples in terms of their balance values. In (b), the stool samples have a distinct bulge near 0, which corresponds to the horizontal line (factor 2) in S19(a) Fig. Furthermore, the third factor in (c) again clearly separates the vaginal samples from the rest, as shown before in S19(c) Fig. (TIFF) [file pone.0217050.s025.tiff]
